# The unique C- and N-terminal sequences of Metallothionein isoform 3 mediate growth inhibition and Vectorial active transport in MCF-7 cells

**DOI:** 10.1186/s12885-017-3355-9

**Published:** 2017-05-25

**Authors:** Brent Voels, Liping Wang, Donald A. Sens, Scott H. Garrett, Ke Zhang, Seema Somji

**Affiliations:** 1 Department of Pathology, University of North Dakota, School of Medicine and Health Sciences, 1301 N. Columbia Road, Stop 9037, Grand Forks, ND 58202 USA; 2 Departments of Science, Cankdeska Cikana Community College, 214 1st Avenue, Fort Totten, ND 58335 USA; 3 Present address: Department of Medical Ultrasound, Tongji Hospital, Tongji Medical College, Huangzhong University of Science and Techology, Wuhan, 430030 People’s Republic of China

**Keywords:** Breast cancer, MT3, MT1E, MCF-7, GAGE genes, PIP6, Dome formation, Vectorial active transport

## Abstract

**Background:**

The 3rd isoform of the metallothionein (MT3) gene family has been shown to be overexpressed in most ductal breast cancers. A previous study has shown that the stable transfection of MCF-7 cells with the MT3 gene inhibits cell growth. The goal of the present study was to determine the role of the unique C-terminal and N-terminal sequences of MT3 on phenotypic properties and gene expression profiles of MCF-7 cells.

**Methods:**

MCF-7 cells were transfected with various metallothionein gene constructs which contain the insertion or the removal of the unique MT3 C- and N-terminal domains. Global gene expression analysis was performed on the MCF-7 cells containing the various constructs and the expression of the unique C- and N- terminal domains of MT3 was correlated to phenotypic properties of the cells.

**Results:**

The results of the present study demonstrate that the C-terminal sequence of MT3, in the absence of the N-terminal sequence, induces dome formation in MCF-7 cells, which in cell cultures is the phenotypic manifestation of a cell’s ability to perform vectorial active transport. Global gene expression analysis demonstrated that the increased expression of the GAGE gene family correlated with dome formation. Expression of the C-terminal domain induced GAGE gene expression, whereas the N-terminal domain inhibited GAGE gene expression and that the effect of the N-terminal domain inhibition was dominant over the C-terminal domain of MT3. Transfection with the metallothionein 1E gene increased the expression of GAGE genes. In addition, both the C- and the N-terminal sequences of the MT3 gene had growth inhibitory properties, which correlated to an increased expression of the interferon alpha-inducible protein 6.

**Conclusions:**

Our study shows that the C-terminal domain of MT3 confers dome formation in MCF-7 cells and the presence of this domain induces expression of the GAGE family of genes. The differential effects of MT3 and metallothionein 1E on the expression of GAGE genes suggests unique roles of these genes in the development and progression of breast cancer. The finding that interferon alpha-inducible protein 6 expression is associated with the ability of MT3 to inhibit growth needs further investigation.

**Electronic supplementary material:**

The online version of this article (doi:10.1186/s12885-017-3355-9) contains supplementary material, which is available to authorized users.

## Background

The metallothioneins (MTs) are a class of low-molecular weight (M_r_ = 6000–7000), cysteine-rich, inducible, intracellular proteins best known for their high affinity to bind heavy metals and mediate cell toxicity [1, 2]. In rodents, there are 4 isoforms of the MT protein designated as MT1 through MT4 that can be characterized on the basis of charge and sequence. These 4 MT isoforms are each encoded by a single gene. The MT1 and MT2 isoforms have been extensively studied for their role in mediating heavy metal toxicity. They have as a hallmark their rapid transcriptional induction in almost all tissues following exposure to metals, such as zinc and cadmium [3]. In the mouse, the genes encoding MT1 and MT2 are approximately 6 kb apart on chromosome 8 and are coordinately regulated and functionally equivalent [4, 5]. Two additional members of the MT gene family have been identified and designated as MT3 and MT4 which are closely linked to, but not coordinately regulated with the other MT genes on mouse chromosome 8 [6, 7]. The MT3 and MT4 family members have not received the extensive study that characterized the MT1 and MT2 isoforms as mediators of cellular toxicity. While humans possess the four major isoforms of MT (1, 2, 3, and 4) that are present in rodents, due to a gene duplication event, the human MT1 locus encodes additional MT1 isoforms that are not present in rodents. In humans, the MTs are encoded by a family of genes located at 16q13 that encode 11 functional and 6 non-functional MT isoforms. The functional MT genes include 8 functional MT1’s (1A, 1B, 1E, 1F, 1G, 1H, 1 M and 1X) and one functional gene for MT2, MT3 and MT4 [8–10]. The human MT1, MT2 and MT4 genes display a very high level of sequence homology, which prevents the generation of an antibody specific for each of the MT1, 2 or 4 isoforms [11]. A mouse monoclonal, anti-horse MT antibody (E9) is commercially available that is easy to use and has been shown to interact with the human MT1, MT2 and MT4 isoforms. This antibody has been used extensively on archival formalin-fixed, paraffin-embedded patient samples to define the immunohistochemical expression of MT1, 2 and 4 in a variety of human cancers [12, 13]. Overall, these studies have shown an association of MT1 and MT2 overexpression with the type and grade of the tumor, with aggressive cancers having the highest levels of MT1/2 expression.

This laboratory is interested in examining the expression of MT3 in human disease since the MT3 isoform has several unique features that distinguish it from the MT1 and MT2 isoforms. The MT3 isoform has a very limited distribution in normal tissues compared to the MT1 and MT2 isoforms and was initially characterized as a brain-specific MT family member [7]. This isoform is not induced by exposure to metals or other factors shown to elicit large increases of gene transcription for the MT1 and MT2 isoforms. The MT3 protein was originally named growth inhibitory factor, but was subsequently renamed MT3 when it was shown to possess many of the characteristic features of the traditional MTs, including transition metal binding [14, 15]. The MT3 isoform has two structurally unique features compared to all other MT family members. It possesses 7 additional amino acids that are not present in any other member of the MT gene family, a 6 amino acid C-terminal sequence and a threonine (Thr) in the N-terminal region [7, 14, 15]. The unique C-terminal sequence has allowed this laboratory to generate a MT3 specific antibody [16]. Functionally, MT3 has been shown to possess a neuronal cell growth inhibitory activity which is not duplicated by the other human MT classes [15, 17]. This non-duplication of function occurs despite a 63–69% homology in amino acid sequence among MT3 and the other human MT isoforms [11]. The neuronal growth inhibitory activity of MT3 has been shown to require the unique N-terminal Thr sequence and not the unique 6 amino acid C-terminal sequence [11]. To date, no function has been assigned to the unique C-terminal sequence of MT3.

The present study was designed to further define the role of MT3 expression in human breast cancer. This laboratory has shown that MT3 mRNA and protein is not expressed in normal human breast tissue [18]. A corresponding immunohistochemical analysis of MT3 expression in a small archival set of patient samples of human breast cancers showed that all breast cancers stained positive for the MT3 protein and that the level of expression was associated with cancers having a poor prognosis. An expansion of this study to a much larger archival set of patient samples showed that few of the breast cancers did not express MT3, but that the absence of MT3 expression was a favorable marker for disease outcome [19]. A high frequency of MT3 staining was also demonstrated for in situ breast cancer, suggesting MT3 might be an early biomarker for disease development. It was also shown in the above study that the MCF-10A breast cell line had no expression of MT3, but the expression could be induced following treatment with a histone deacetylase inhibitor and that the MT3 metal regulatory elements were potentially active binders of transcription factors following treatment. In addition, the laboratory has shown that the MCF-7 breast cancer cell line does not express MT3 and that stable transfection and expression of the MT3 gene inhibits the growth of the MCF-7 cells. The expression of MT3 in breast cancer has also been observed in other studies [20–22] and in triple negative breast cancers, it has been suggested that its expression is associated with poor prognosis [22]. In pediatric acute myeloid leukemia, the promoter of the MT3 gene is hypermethylated suggesting that it may function as a tumor suppressor [23].

The goal of the present study was to determine the role of the C-terminal and N-terminal sequences of MT3 on phenotypic properties and gene expression profiles of MCF-7 cells.

## Methods

### Cell culture

The MCF-7 cell line (Cat. No. ATCC® HTB22™) was obtained from the American Type Culture Collection (Rockville, MD), grown in Dulbecco’s Modified Eagles’ medium supplemented with 5% (*v*/v) fetal calf serum, and routinely passaged at a 1:4 ratio upon attaining confluence. Growth curves were generated following subculture of confluent cultures of wild type MCF-7 cells and their stable transformants at a 1:100 ratio into six-well plates. The increase in cell growth was determined every 24 h by measuring the capacity of the cells to reduce MTT (3-(4,5-dimethylthiazol-2-yl)-2,5-diphenyltetrazolium bromide) to formazan [24]. The absorbance was determined at 570 nm using a plate reader with acidic propanol as the blank. Triplicate cultures were analyzed at each time point and doubling times calculated from the linear region of the exponential portion of the growth curve.

### Stable transfection of MCF-7 cells

The various gene constructs that were made by the alteration of the unique MT3 N- and C-terminal region have been described in detail previously [25]. These constructs were stably transfected into the MCF-7 cells and are designated as wild type MT3 (MT3), MT3 with an N-terminal mutation where the two essential prolines were converted to threonines (MT3ΔNT), MT3 with a C-terminal deletion where the unique EAAEAE C-terminal sequence was deleted (MT3ΔCT), wild type MT1E (MT1E), MT1E where the MT3 N-terminal sequence was inserted into the corresponding position of MT1E (MT1E-NT), and MT1E where the C-terminal sequence EAAEAE of MT3 was inserted into the corresponding position of MT1E (MT1E-CT). The constructs were blunt end ligated into the 6.2/V5 Destination vector (Invitrogen, NY) and were linearized using BspHI (New England Biolabs, MA) prior to transfection using the Effectene reagent (Qiagen, CA). Sequence design for ligation was done utilizing the Vector NTI® computer software (Life Technologies, NY). Generation of the mutant sequences and ligation of the genes was conducted by GenScript (Piscataway, NJ) using the wild type MT3 gene sequence. Plasmids were transformed using One Shot® TOP10/P3 *E. coli* cells (Life Technologies, NY) and purified using a Qiagen midi prep kit (Qiagen, CA). Transfected cells were allowed to reach confluency in one well of a 6-well plate and then sub-cultured at a 1:10 ratio into a 6-well plate. Transfected cells were propagated in media containing 10 μg/mL blasticidin (Invitrogen, CA). Selected colonies were expanded and harvested for RNA isolation. Positive clones were expanded and used for downstream applications.

### Real-time PCR and Western blot analysis

The level of expression of mRNA from the MCF-7 cells transfected with wild type MT3 and the various C- and N-terminal mutations was determined using specific primers to the V5 region of the expression vector. The sequences of the primers are: forward 5- TTCGAAGGTAAGCCTATCCCT -3 and reverse 5- AGTCATTACTAACCGGTACGC -3. The primers used for the GAGE antigen were obtained from Qiagen and are as follows: GAGE2C (Cat no. QT01001035), GAGE2E-1 (Cat no. QT01018696), GAGE2E-2 (Cat no. QT01672202), GAGE4 (Cat no. QT00197015), GAGE5 (Cat no. QT01001042), GAGE6 (Cat no. QT01001049), GAGE12G (Cat no. QT01530627) and GAGE12H (Cat no. QT01664495). Real-time PCR was performed utilizing the SYBR Green kit (Bio-Rad, CA) with 2 μl of cDNA, 1 μl primers in a total volume of 20 μl in CFX real-time detection system (Bio-Rad, CA). The denaturation was performed at 94 °C, followed by annealing at 60 °C and extension at 72 °C. The amplification was monitored by SYBR Green fluorescence. The data was compared with that of a standard curve consisting of serial dilutions of cDNA from the pcDNA 6.2/V5 transfected cells. The expression of mRNA for the G antigen (GAGE) genes was assessed using gene-specific primers (Bio-Rad, CA). GAGE gene expression is expressed as fold change compared to the MCF-7 cells tranfected with the blank pcDNA 6.2/V5 vector. Western blot analysis of the GAGE gene family was performed using protocols described previously [26]. The primary GAGE7 antibody was purchased from Thermo Fisher Scientific (Rockford, IL).The antibody was made against amino acids 87–116 of the C-terminal region of human GAGE7. A blast search has shown that this sequence is present in all GAGE isoforms and can detect all isoforms of the GAGE protein. The blots were visualized using Clarity Western ECL (Bio-Rad Laboratories).

### Dome formation by MCF-7 cell lines

The various MCF-7 cell lines were grown in triplicates in T-25 flasks. Cells were fed fresh growth media every three days and cultures were observed for dome formation at confluency. A dome is defined microscopically when a group of cells appears out-of-focus in relation to the in-focus monolayer, and conversely when the dome is in-focus, the rest of the monolayer appears out-of-focus. The number of domes in a field of view was determined for each culture and a field of view is defined by the area examined through a 100× field of view. Twenty-one field of views were observed for each T-25 culture flask.

### Transepithelial resistance

Measurement of transepithelial resistance (TER) was performed as described previously [27]. Briefly, cells were seeded at a 2:1 ratio in triplicate onto 30 mm diameter cellulose ester membrane inserts (Corning, NY) placed in six-well trays. Beginning on the fifth day post-seeding, TER was measured on day 5, 6 and 7 with the EVOM Epithelial Voltohmmeter (World Precision Instruments, Sarasota, FL) with a STX2 electrode set according to the manufactures instructions. The resistance of the bare filter containing medium was subtracted from that obtained from filters containing cell monolayers. Two sets of four readings were taken at two different locations on each filter. Parallel cultures of the cells were also monitored for dome formation. The experiment was performed in triplicates and the final result reported as the mean ± SE.

### Preparation of RNA for microarray analysis

The Qiagen RNeasy Mini Kit was used to prepare RNA samples from the various MCF-7 cell lines for use in microarray analysis. RNA was harvested from confluent cultures of cells during periods where dome formation was present in cultures previously shown to form domes. The cells were lysed in RLT buffer containing β-mercaptoethanol. The QiaShredder column was used to homogenize the lysates and the RNA was isolated following the manufacturers protocols.

### Microarray analysis

RNA samples were sent to the University of Minnesota Genomics Center for microarray analysis. The Human HT-12v4 Expression BeadChip (Illumina, CA) was utilized to determine genome wide gene expression levels. The Bioinformatics core facility at the University of North Dakota School of Health and Medicine Sciences analyzed the resulting data for differentially expressed genes. Differentially expressed probe sets (DEGs) were identified using Significance Analysis of Microarrays (SAM) method [28] and the *p*-values were adjusted using false discovery rate. The analyses were carried out using R programming language.

A new clustering method, overlap hierarchical clustering (OHC) was developed to assess the similarity and variation across isolates. In order to reflect the gene expression changes, a new dissimilarity measure, overlap distance, was introduced to hierarchical clustering. Overlap distance measures are based on the number of genes that have large fold changes in both transformed cell lines comparing with parental MCF-7 cells. The fold change of each probe in each array from a transformed cell line was calculated over its average expression level in the parental MCF-7 cell line. If the fold change was greater than 2 in the transformed cell line A, the probe was selected for the gene set A. The overlap distance between cell lines A and B was calculated as follows:

$$ D\left( A, B\right)=1-\frac{\mid \mathrm{A}\cap \mathrm{B}\mid }{\mid \mathrm{A}\cup \mathrm{B}\mid } $$.

The distance between two clusters was calculated by Ward’s linkage method.

### Statistics

All experiments were performed in triplicates and the results are expressed as the standard error of the mean. Statistical analyses were performed using GraphPad Prism® software using separate variance t-tests, ANOVA with Tukey post-hoc testing.

## Results

### Measurement of dome formation, an indicator of vectorial active transport in MCF-7 cells

**Fig. 1:**
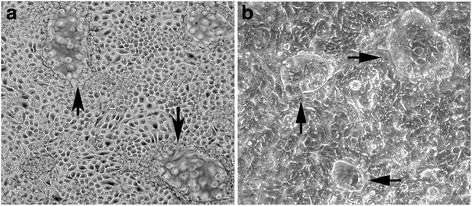
Light level morphology of domes. **a**. Dome formation in human proximal tubule cells. **b**. Dome formation in MCF-7 cells expressing the C-terminal domain of MT3 (MT1E-CT). Arrows indicate the presence of domes (both at 100× magnification)

**Fig. 2:**
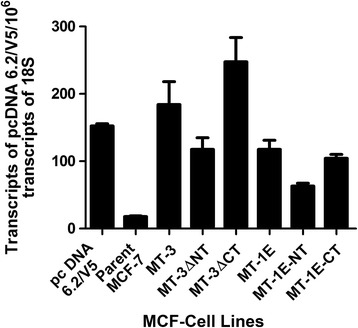
Expression of MT3 mutants in MCF-7 cells. Real time PCR analysis was performed to determine the expression of the pcDNA 6.2/V5vector through the amplification of the common V5 sequence in the 3 prime end of the expressed sequence. The results are expressed per 10^6^ transcripts of 18S ribosomal RNA. The data is plotted as the mean ± SEM of 3 independent determinations

Domes are a hallmark of cultured epithelial cells that retain the in situ property of vectorial active transport [29–31]. As detailed in these reports, these out-of-focus areas of the cell monolayer seen upon light microscopic examination represent raised areas where fluid is trapped underneath the monolayer owing to active transport of ions and water across the cell monolayer in an apical to basolateral direction. This in turn traps a bubble of fluid between the cell layer and the culture dish, forcing local detachment of the monolayer from the plastic surface forming a raised area with an underneath reservoir of accumulated fluid. The three requirements for dome formation by a cell is the presence of basolateral Na^+^,K^+^-ATPase, apical tight junctions and electrogenic active transport. There is no evidence in our study that wild type MCF-7 cells form domes in cell culture. An unexpected result in the present study was the finding that MCF-7 cells stably transfected with selected MT gene constructs containing the C-terminal domain of MT3 gained the ability to form domes. In the present study, the number of domes in a 100× microscopic field was used to quantify dome formation by the stably transfected MCF-7 cell lines. To illustrate the structure counted, a typical dome formed by transporting renal epithelial cells is shown at 100× magnification for a human proximal tubule cell culture from this laboratory [31], as well as one from a MCF-7 cell line expressing the C-terminal domain of MT3 (MT1E-CT), both at 100× magnification (Fig. 1a and b). There were 2 experimental conditions where the MCF-7 cells gained the ability to form domes (Table 1). The first was when the MCF-7 cells were stably transfected with the MT1E gene modified to contain the C-terminal sequence of MT3 (MT1E-CT). The second was when the MCF-7 cells were stably transfected with the MT3 gene sequence with a mutated N-terminal domain (MT3ΔNT). The MCF-7 cells stably transfected with the wild type MT3 (MT3) formed very few small domes. Real time PCR was performed on each stably transfected MCF-7 cell line to confirm the expression of the constructs and the results showed that each construct was expressed as expected in each of the respective MCF-7 cell lines (Fig. 2).

**Table 1:** Number of domes observed in various MCF-7 MT3 mutants

MCF-7 Cell Lines	Domes observed	Average number of domes observed per field of view	Average number of domes observed per 21 fields of view
MT3	Yes/No	0.1	2.1
MT3ΔCT	Yes	2.72	57.22
MT3ΔNT	No	0	0
MT1E	No	0	0
MT1E-CT	Yes	2.69	56.44
MT1E-NT	No	0	0
MCF-7 (parent)	No	0	0
pcDNA 6.2/V5 Blank vector	No	0	0

The TERs of monolayer cultures of the parental MCF-7 cell line and their stably transformed counterparts were measured on days 5, 6 and 7 after the cells attained confluence. Transepithelial resistance is an established method to determine the presence of tight junctions between cells along with the cells ionic permeability. The results demonstrated that all the MCF-7 cell lines generated a measurable TER of similar magnitude (Table 2). This level of TER would be indicative of a cell line having tight junctions between cells, but with a high permeability to ion movement and it would be classified as a monolayer with “leaky tight junctions”. Thus, these results suggest that the C- and N-terminal domain have no influence on TER, since the TER did not change when the MCF-7 cells were transfected with any of the constructs.

**Table 2:** TERs measured in various MCF-7 MT3 mutants

MCF-7 Cell Lines	Average TER Day 5 (Ω/cm^2^)	Average TER Day 6 (Ω/cm^2^)	Average TER Day 7 (Ω/cm^2^)	Average TER Day 5, 6 and 7 (Ω/cm^2^)
MT3	35.32 +/− 5.81	53.57 +/− 13.88	52.27 +/− 12.11	48.72
MT3ΔCT	41.99 +/− 9.26	53.08 +/− 9.57	47.67 +/− 5.16	47.58
MT3ΔNT	26.39 +/− 1.99	44.06 +/− 3.37	28.16 +/− 5.87	32.87
MT1E	32.68 +/− 7.26	35.92 +/− 6.98	30.91 +/− 8.41	33.17
MT1E-CT	22.96 +/− 11.94	29.34 +/− 9.40	37.98 +/− 7.07	30.09
MT1E-NT	37.58 +/− 10.23	34.3 +/− 9.4	32.28 +/− 12.61	34.72
MCF-7 (parent)	38.80 +/− 12.10	22.21 +/− 4.63	32.4 +/− 1.92	31.13
pcDNA 6.2/V5 Blank vector	41.99 +/− 5.38	27.38 +/− 10.76	36.80 +/− 6.48	35.39

Average TER for each mutant cell line was measured on days 5, 6 and 7 and is expressed as Ω/cm^2^ +/− the SEM. The combined average TER for days 5, 6 and 7 was used to determine the relationship between TER and the presence or absence of the N- and C- terminal region of MT3. No statistical significance using the one-way ANOVA test and Dunnet’s post-test for multiple comparisons using the pcDNA 6.2/V5 cell line as control

### Effect of MT-3 C- and -N terminal sequence alteration on gene expression patterns in MCF-7 cells

**Fig. 3:**
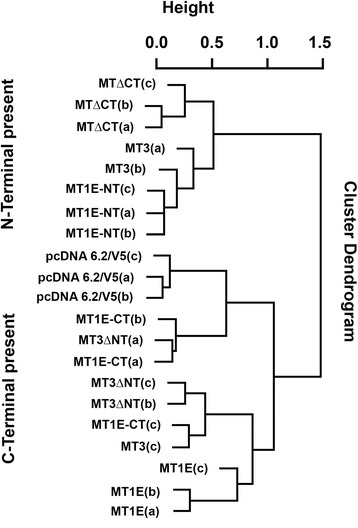
Dendogram showing the relatedness of global gene expression patterns among MCF-7 cells stably expressing each metallothionein construct. Constructs contain either wild-type MT3, MT3 with the N-terminal domain mutated (MT3ΔNT), the C-terminal domain mutated (MT3ΔCT), wild type MT1E, MT1E containing the N-terminal domain of MT3 (MT1E-NT), MT1E with the C-terminal domain of MT3 (MT1E-CT), or the blank vector (pcDNA 6.2/V5). The effect of each construct was assessed in triplicate with each triplicate shown individually as either (*a*), (*b*), or (*c*). The clustering was assessed by overlap hierarchical clustering

Total RNA was isolated from triplicate samples of the wild type MCF-7 cells and the constructs and the samples were subjected to global gene expression analysis employing the Illumia human HT-12v4 expression bead chip. The relationship of the resulting gene expression patterns among all the samples was assessed using the overlap hierarchical clustering (OHC) method. This analysis allowed an initial assessment of the overall relationship of global gene expression patterns to the presence of the two unique domains of MT3, the C-terminal and the N-terminal domains. The results of this analysis demonstrated that the relationship in overall gene expression patterns among all the RNA samples is highly dependent on the presence or absence of the C- and N-terminal domains of the MT3 molecule (Fig. 3). The RNA samples from transfectants possessing the N-terminal domain resided in the upper cluster of the dendrogram and those possessing the C-terminal domain resided in the low cluster of the dendrogram. The triplicate isolates of MCF-7 cells stably transfected with the MT3 wild type gene were split between the two clusters, with 2 of the 3 isolates in the upper N-terminal cluster of the dendrogram and the remaining isolate in the lower C-terminal cluster. The segregation of the triplicate wild type MT3 MCF-7 cells into the two clusters renders it unclear which domain of the MT3 molecule exhibits dominant activity.

### Correlation of global gene expression profiles and the induction of dome formation by MT3 C-terminal sequences in stably transfected MCF-7 cells

The ability of the MT1E gene, when modified to contain the C-terminal sequence of MT3, to induce dome formation by MCF-7 cells provides a phenotypic alteration that can be correlated to global gene expression profiles. Three paired comparisons were analyzed to determine potential correlations between dome formation and the C- and N-terminal domains of MT3. The first was a comparison of MCF-7 transfected by the wild type MT1E gene (MT1E) with that of the cells transfected with MT1E modified to contain the C-terminal sequence of MT3 (MT1E-CT). The second was a comparison of MCF-7 cells transfected with MT1E compared with that of the cells transfected with MT1E modified to contain the N-terminal sequence (MT1E-NT). The final comparison was the MCF-7 cells transfected with the wild type MT3 gene (MT3) compared with that of cells transfected with the MT3 gene with a mutated N-terminal sequence (MT3ΔNT). The results of these comparisons are presented in Additional files 1, 2 and 3 respectively.

The results of the paired comparisons with one another demonstrates a strong correlation of GAGE family gene expression with the ability of the MCF-7 cells to form domes. GAGE family genes were up-regulated and the MCF-7 cells were able to dome when the MCF-7 cells were transfected with the MT1E gene containing the C-terminal sequence of MT3 (MT1E vs MT1E-CT, Additional file 1) and when the MCF-7 cells were transfected with an MT3 construct containing a mutated N-terminal sequence (MT3 vs MT3ΔNT, Additional file 3). In contrast, the GAGE family of genes were down-regulated and the cells did not form domes, when the MCF-7 cells were transfected with MT1E containing the N-terminal sequence of MT3 (MT1E vs MT1E-NT, Additional file 2). Thus, the paired comparisons implicate the GAGE family of genes in the ability of the C-terminal sequence of MT3 to induce dome formation in MCF-7 cells transfected with the MT1E or MT3 gene.

### Validation of GAGE gene expression in MCF-7 cells transfected with C- and N-terminal sequence of MT3

**Fig. 4:**
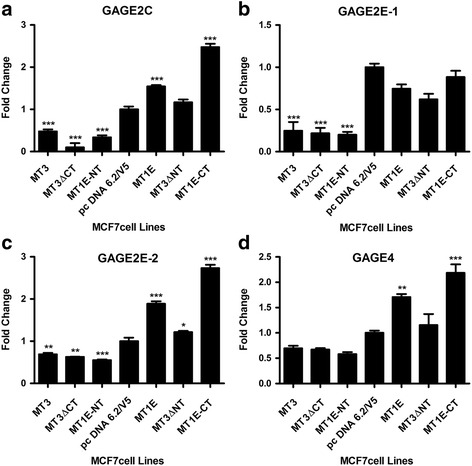
Expression of GAGE genes in MCF-7 cells transfected with various MT3 mutants. Real time PCR analysis of GAGE2C (**a**), GAGE2E-1 (**b**), GAGE2E-2 (**c**) and GAGE4 (**d**) genes. The results are expressed as fold change compared to the vector pcDNA 6.2/V5. *denotes significantly different from vector control (*p* < 0.05). **denotes significantly different from vector control (*p* < 0.01). ***significantly different from vector control (*p* < 0.001). The data is plotted as the mean ± SEM of 3 independent determinations

**Fig. 5:**
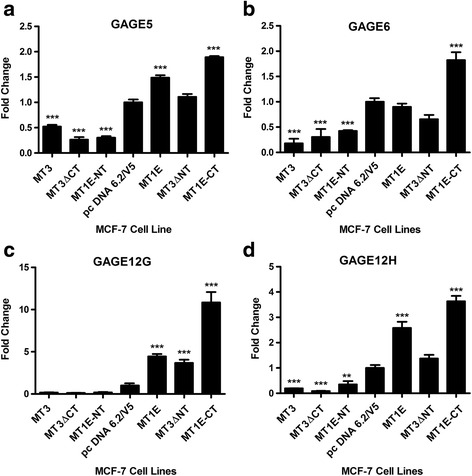
Expression of GAGE genes in MCF-7 cells transfected with various MT3 mutants. Real time PCR analysis of GAGE5 (**a**), GAGE6 (**b**), GAGE12G (**c**) and GAGE12H (**d**) genes. The results are expressed as fold change compared to the vector pcDNA 6.2/V5. **denotes significantly different from vector control (*p* < 0.01). ***significantly different from vector control (*p* < 0.001). The data is plotted as the mean ± SEM of 3 independent determinations

Based on the results of the above microarray comparison, the expression of the GAGE family of genes was confirmed using real-time PCR. Due to sequence homology, the genes that were validated were: GAGE2C; GAGE2E-1; GAGE2E-2; GAGE4; GAGE5; GAGE6; GAGE12G; and, GAGE12H. GAGE12F was not validated since a suitable primer sequence could not be identified for use. Several general patterns of gene expression were observed for the GAGE gene family (Figs 4 and 5). The first was when total RNA from MCF-7 cells carrying a blank vector control (pc DNA 6.2/V5) was analyzed against total RNA from the WTMT3, MT3ΔCT and, MT1E-NT cell lines. The results from this analysis showed that all the three cell lines had significantly lower expression of GAGE2C, GAGE2E-1, GAGE2E-2, GAGE5, GAGE6 and GAGE12H genes and there was a trend for reduced expression of the GAGE4 and CAGE12 genes. A second pattern of expression was found when GAGE gene expression was compared between the blank vector control and the MT1E cell lines. In this analysis, the expression of 6 of the 8 GAGE family members was increased in MCF-7 cells stably transfected with the MT1E gene (GAGE2C, GAGE2E-2, GAGE4, GAGE5, GAGE12G, GAGE12H). The remaining 2 GAGE genes (GAGE2E-1, GAGE6) showed no difference in expression. In addition, 7 of the 8 GAGE genes were also increased when MT1E-CT was compared to the blank vector control or the MT1E construct, the exception being the GAGE2E-1 gene. Finally, confirming the results of the above microarray analysis, all the MCF-7 cell lines containing an N-terminal sequence (MT3, MT3ΔCT, MT1E-NT) had reduced expression of all the GAGE genes when compared to the MCF-7 cell lines containing a C-terminal sequence (MT3ΔNT, MT1E-CT) or MT1E.

**Fig. 6:**
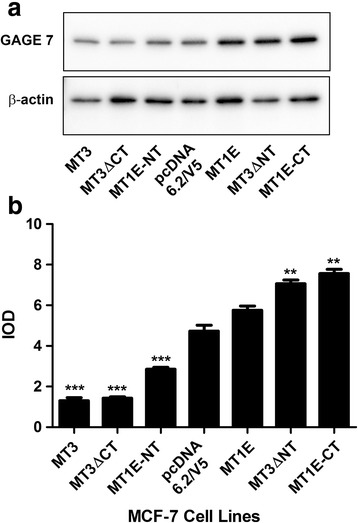
Western blot analysis of GAGE gene expression in MCF-7 cells transfected with various MT3 mutants. (**a** and **b**). The integrated optical density (IOD) of each band was normalized to the IOD of β-actin. **denotes significantly different from vector control (*p* < 0.01). *** Significantly different from vector control (*p* < 0.001).The data is plotted as the mean ± SEM of 3 independent experiments. The image shown is representative of one of the three Western blots performed

The GAGE gene family displays a very high sequence homology, which has prevented the generation of antibodies against the individual GAGE family members. A polyclonal antibody that recognized multiple members of the GAGE family is available. This antibody was used in Western blot analysis to determine the combined expression of the GAGE family proteins (Fig. 6). The results showed an overall trend of GAGE protein expression that followed the mRNA expression pattern for the individual GAGE genes, that is, all the MCF-7 cell lines containing an N-terminal sequence (MT3, MT3ΔCT, MT1E-NT) had reduced expression of the GAGE proteins when compared to the MCF-7 cell lines containing a C-terminal sequence (MT3ΔNT, MT1E-CT) or MT1E. There was a decrease in expression of GAGE proteins in the MCF-7 cells containing the MT3ΔCT and MT1E-NT constructs when compared to the cells expressing the blank vector pcDNA 6.2/V5, whereas the cells containing the MT3ΔNT and MT1E-CT constructs showed significant increases in GAGE protein expression when compared to the cells expressing the blank vector pcDNA 6.2/V5. The fact that the antibody recognizes the protein from multiple GAGE family members limits the significance of the findings to individual family members.

### Correlation of global gene expression profiles and the inhibition of cell growth by MT3 C-terminal and N-terminal sequences in stably transfected MCF-7 cells

**Fig. 7:**
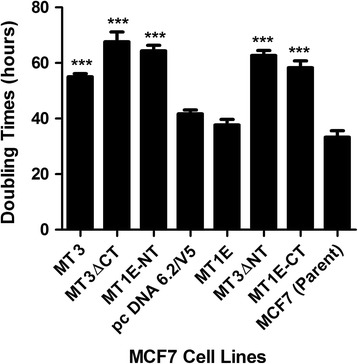
Doubling times of MCF-7 cells transfected with various MT3 mutants. The doubling times of the transfected cells were compared to that of the blank vector control pcDNA 6.2/V5. ***significantly increased compared to pcDNA 6.2/V5 (*p* < 0.001). The data is plotted as the mean ± SEM of 3 independent determinations

As detailed in the introduction, the laboratory has previously shown that stable transfection of MCF-7 cells with the MT3 coding sequence inhibits the growth of the MCF-7 cell line. The doubling times of MCF-7 cells in their logarithmic growth phase was determined for wild type MCF-7 cells and MCF-7 cells stably transfected with the various constructs containing the addition and deletions of the C- and N-terminals. The results showed that the wild type MCF-7 cells (Parent), MCF-7 cells stably transfected with the MT1E coding sequence (MT1E), and MCF-7 cells stably transfected with a blank vector control had similar doubling times (Fig. 7). The doubling times were 32.5 ± 4.4, 35.8 ± 4.7 and 39.5 ± 5.9 h respectively. In contrast, the MCF-7 cells stably transfected with MT3, MT3ΔNT, MT3ΔCT, MT1E-NT, and MT1E-CT all displayed significantly higher doubling times (Fig. 7). The doubling times were 53.1 ± 2.2, 57.3 ± 3.8, 64.7 ± 5.2, 60.9 ± 3.3, and 55.2 ± 11.2 h, respectively. There were no significant differences of doubling times within the members of each of the two groups. These results indicate that both the C-terminal and N-terminal sequences of MT3 reduce the rate of growth of MCF-7 cells.

In order to determine if the mechanism of action involved in the growth inhibition elicited by the C- and N-terminal domains were similar, the global gene expression profiles were examined and a comparison was made between MCF-7 cells transfected with MT1E versus MT1E-CT and MT1E-NT, respectively (Additional files 1 and 2). The results demonstrated that there were 5 genes common to both sets. Phosphoglucomutase-like protein 5 (PGM5) and insulin like growth factor binding protein 5 (IGFBP5) were upregulated whereas interferon alpha-inducible protein 6 (IFI6), DnaJ heat shock protein family (Hsp40) member C12 (DNAJC12) and protein S (alpha) (PROS1) were downregulated in MT1E-CT and MT1E-NT. The expression of these genes were then determined in the other sets that also showed reduced growth rates. A comparison was made between the MCF-7 cells (blank vector control) versus MT3 (Additional file 4), MT3ΔCT (Additional file 5), and MT3ΔNT (Additional file 6). The only gene common among the 5 sets of comparisons that correlated to reduced cell growth was the down-regulation of IPI6 in cells containing the C- or N-terminal sequence of MT3.

## Discussion

As detailed in the introduction, this laboratory has shown that stable transfection of MCF-7 cells with MT3 results in the inhibition of cell growth. The original goal of the present study was to determine if the unique N-terminal sequence of MT3 was necessary for the inhibition of MCF-7 cell growth, similar to that found for the N-terminal sequence in the neural system [11]. The strategy employed involved the stable transfection of the MCF-7 cells with various MT constructs deleting or adding the unique C- and N-terminal sequences of MT3. The human MT1E gene was chosen as the vector for transfection of the MCF-7 cells with additions of the unique C- and N-terminal sequences of MT3 because this laboratory has previously shown that the MT1E gene is not expressed in MCF-7 cells [32]. The results of these stable transfections, coupled with an analysis of global gene expression profiles, provided several new insights on the contributions of the C- and N-terminal sequences to the function of MT3 well beyond the possible role of the N-terminal sequence in the inhibition of MCF-7 cell growth.

A unique finding in the present study was the elucidation of an MCF-7 cell phenotype that could be correlated with the C-terminal sequence of MT3. This cell phenotype was the ability of the MCF-7 cells to form domes in culture, a manifestation of vectorial active transport, a process that requires electrogenic active sodium transport, a functional Na^+^,K^+^-ATPase and apical tight junctions between cells. The results demonstrated very convincingly that MCF-7 cells transfected with the MT1E gene, modified to contain the C-terminal sequence of MT3, gained the ability to form domes in culture. It was also demonstrated that MCF-7 cells transfected with MT3 having a mutated N-terminal sequence, but containing an unmodified C-terminal sequence, also allowed the cells to form domes in culture. Overall, the stable transfection strategy showed that the presence of the C-terminal sequence, in the absence of the N-terminal sequence, allowed MCF-7 cells to gain the function of vectorial active transport. However, when the N-terminal sequence was present it was dominant over the C-terminal sequence and the ability to induce vectorial active transport was inhibited in the MCF-7 cells. The series of stable transfectants was subjected to global gene expression analysis and the results suggested that an increase in the expression of the GAGE gene family was correlated with the ability of the C-terminal sequence to induce dome formation and the N-terminal sequence in preventing dome formation. However, the differences in global gene expression patterns were not large and the results were successfully validated by real-time PCR for the GAGE2C; GAGE2E-1; GAGE2E-2; GAGE4; GAGE5; GAGE6; GAGE12G; and GAGE12H family members. The results of the validation were consistent with the N-terminal sequence of MT3 suppressing the expression of the GAGE gene family in MCF-7 cells, and when absent, with the ability of the C-terminal sequence to induce GAGE gene expression in the cells. Due to the extensive sequence homology between members of the GAGE gene family, the antibody used for this study cross-reacts with several of the family members and the data obtained from the Western blot analysis showed overall GAGE protein expression in agreement with the mRNA expression of the individual GAGE family members.

There is only limited information available on the GAGE gene family. The GAGE antigens are a member of the cancer/testis (CT) antigen group of proteins expressed only in germ cells of healthy individuals. Currently there are eighty-nine CT antigens all of which are encoded on the X chromosome [33]. The GAGE antigens are a family of CT antigens consisting of 13 to 39 copies of nearly identical genes on chromosome x at p11.23 [34]. The promoters of the GAGE antigen family have no TATA box, and have only one or two different base pairs in the first fourteen hundred base pairs of the promoter [33]. The lack of a TATA box site for initiation allows transcription to start from several different sites leading to transcripts of varying lengths [35]. The exact biological function of the GAGE antigens is unknown, but recent evidence suggests that they may direct cell proliferation, differentiation, and the survival of germ line cells [36]. Anti-apoptotic properties have been attributed to GAGE antigens [35]. Expression of the GAGE antigens normally occurs in a subset of oocytes in the adult ovary [37], adult male germ cells, and for a few weeks in fetal Leydig and Sertoli cells during the third trimester [38].

Despite the very limited distribution of GAGE antigens in the germ cells of healthy individuals, they have been found to gain expression in a variety of human cancers. The expression of GAGE antigens in stomach cancer, neuroblastoma, and esophageal carcinoma has been correlated with a poor prognosis and aggressive tumor type [39–41]. The activation of the GAGE antigens in a variety of cancers, as well as the cancer/testis antigens in general, has been the subject of a recent review [42]. Important to the current study is that two studies do show an alteration of GAGE gene expression in breast cancer [37, 43]. The first showed an increase in GAGE gene transcripts in 26% of breast cancers and the second, in 17% of breast cancers. The expression of GAGE was localized primarily in the cytoplasm with rare profiles of nuclear localization. Moderate expression was found in 9 of 54 tumor samples and strong staining in 8 of the 54 cases. GAGE expression was negative in grade 1 tumor samples with positivity restricted to grade 2 and 3 tumors. There was a trend for, but not a statistically significant, negative effect of GAGE expression on disease-free survival and overall survival [43]. These findings are important for the present study since the expression of MT3 in the MCF-7 cell line inhibits the expression of the GAGE genes. Further studies to define the expression of the GAGE proteins in breast cancer and the mechanism by which MT3 inhibits GAGE gene expression in MCF-7 cells are currently hindered by the lack of antibodies specific to the individual GAGE family members. In addition, the high degree of sequence homology within the family and the lack of a TATA box in the promoter may further complicate the generation of GAGE specific reagents.

A second interesting and unexpected finding in the present study was that GAGE gene expression increased when the MCF-7 cells were stably transfected to express the MT1E isoform. The MT1E gene was chosen as a vector in the present study to determine the effect of the unique C- and N-terminal sequences of MT3 since it is not expressed in the MCF-7 cell line [32]. However, the MCF-7 cell line does express other isoforms as the MT2A and MT1X genes have been shown to have basal expression [32]. The induction of GAGE gene expression by the MT1E isoform is interesting since there is some evidence that the expression of MT1E is altered in breast cancer and breast cancer cell lines. The above referenced study that showed MT1E not being expressed in MCF-7 cells also showed that the expression of MT1E was absent in an additional estrogen receptor positive cell line T-47D. In contrast, both Hs578T and MDA-MB-231, which are estrogen receptor negative cell lines, were shown to express the MT1E isoform. These results suggested a possible relationship between estrogen receptor status and MT1E gene expression. Evidence that this finding might translate to human specimens of breast cancer tumors is provided by a study on a series of fresh breast cancers which showed that the MT1E isoform was highly expressed in estrogen receptor negative compared to estrogen receptor positive breast cancers [44]. Exploring a potential relationship between the GAGE gene family and the MT1 and MT2 gene family would be of interest, since the expression of MT1/2 has been studied extensively decades ago in ductal breast cancer. The overexpression has been shown to occur early in the disease and is associated with the more malignant, higher-grade tumors, and therefore with poor patient prognosis [45–51]. The expression of MT1/2 has been shown to predict resistance to tamoxifen [52]. The literature suggests that there is no marker that is more consistently elevated in human cancer, and that is also associated with a poor prognosis than MT1/2 [13]. To the authors’ knowledge there has been no study in other breast cancer cell lines or tissues on the relationship between MT and GAGE gene expression.

The final interesting finding in the present study was an extension of the laboratory’s earlier study that showed MT3 expression decreased that growth of MCF-7 cells [53]. The stable transfection of the MCF-7 cells with the MT1E gene modified to contain either the C- or N-terminal unique sequence of MT3 elicited a decrease in cell growth similar to that noted for MCF-7 cells stably transfected with MT3. Similarly, the stable transfection of MCF-7 cells with MT3 modified to have a deletion of either the C- or N-terminal sequence produced an identical inhibition of cell growth to that of cells transfected with wild type MT3. To the author’s knowledge this is the first time the C-terminal sequence of MT3 has been associated with the inhibition of cell growth. The previous study in the neural system implicated only the N-terminal sequence in growth inhibition [11]. A consequence of this finding is that both the C- and N-terminal sequences of MT3 would have to be rendered inactive to remove the ability of MT3 to inhibit cell growth. As detailed in the results, global expression patterns showed that the only gene that correlated to the ability of MT3 to inhibit the growth of MCF-7 cells was IPI6. This gene also known as G1P3 or IFI-6-16 is suggested to play a role in the regulation of apoptosis [54]. Although information about the function of the protein and its tissue distribution is limited, there is one study which shows that overexpression of this gene confers survival advantage to estrogen receptor positive breast cancers and confers tamoxifen resistance [55]. In addition, this study also suggests that the anti-apoptotic activity of IFI6 has a more pronounced effect on adverse outcomes in estrogen receptor positive breast cancers. Although the role of IFI6 in slowing the growth of MT3 expressing breast cancers is not known, the fact that it is overexpressed will provide a starting point to define the mechanism underlying MT3’s ability to inhibit the growth of MCF-7 cells.

## Conclusions

In conclusion, our study shows that the C-terminal domain of MT3 confers dome formation in the MCF-7 breast cancer cells, whereas both the N-and the C-terminal domain of the molecule can confer growth inhibition in MCF-7 cells. The presence of the C-terminal domain of MT3 induced the expression of the GAGE family of genes whereas the N-terminal domain inhibited the expression of the GAGE genes. The differential effect of MT3 and MT1E on the expression of GAGE genes suggests unique roles of these genes in the development and progression of breast cancer. The finding that IFI6 expression is associated with the ability of MT3 to inhibit growth needs to be investigated further to determine the associated mechanism.

## Additional files

Additional file 1: Differential Expression Profile of MCF-7 Cells Transfected with MT1E or MT1E-CT. Table comparing gene expression profiles of MCF-7 cells transfected with the MT1E gene with MCF-7 cells transfected with MT1E-CT construct. (DOC 224 kb)

Additional file 2: Differential Expression Profile of MCF-7 Cells Transfected with MT1E or MT1E-NT. Table comparing gene expression profiles of MCF-7 cells transfected with MT1E gene with MCF-7 cells transfected with MT1E-NT construct. (DOC 35 kb)

Additional file 3: Differential Expression Profile of MCF-7 Cells Transfected with MT3 or MT3ΔNT. Table comparing gene expression profiles of MCF-7 cells transfected with the MT3 gene with MCF-7 cells transfected with MT3ΔNT construct. (DOC 28 kb)

Additional file 4: Differential Expression Profile of MCF-7 Cells Transfected with MT3. Table comparing gene expression profiles of MCF-7 cells transfected with MT3 gene with MCF-7 cells transfected with pcDNA 6.2/V5 blank vector. (DOC 126 kb)

Additional file 5: Differential Expression Profile of MCF-7 Cells Transfected with MT3ΔCT. Table comparing gene expression profiles of MCF-7 cells transfected with pcDNA 6.2/V5 blank vector with MCF-7 cells transfected with MT3ΔCT construct. (DOC 698 kb)

Additional file 6: Differential Expression Profile of MCF-7 Cells Transfected with MT3ΔNT. Table comparing gene expression profiles of MCF-7 cells transfected with pcDNA 6.2/V5 blank vector with MCF-7 cells transfected with MT3ΔNT construct. (DOC 28 kb)

## Abbreviations

C/T antigen: Cancer/testis antigen
DEGs: Differentially expressed probe sets
DNAJC12: DnaJ heat shock protein family (Hsp40) member C12
GAGE: G antigens
IFI6: Interferon alpha-inducible protein 6
IGFBP5: Insulin like growth factor binding protein 5
MT: Metallothionein
MT1E-CT: MT1E containing the C-terminal region of MT3
MT1E-NT: MT1E mutated to contain the N-terminal region of MT3
MT1E-NT-CT: MT1E mutated to contain the C- and N-terminal of MT3
MT3ΔCT: MT3 with a C-terminal deletion
MT3ΔNT: MT3 with an N-terminal mutation
MTT: 3-(4,5-dimethylthiazol-2-yl)-2,5-diphenyltetrazolium bromide
OHC: Overlap hierarchical clustering
PGM5: Phosphoglucomutase-like protein 5
PROS1: Protein S (alpha)
SAM: Significance analysis of microarrays
Thr: Threonine, TER: transepithelial resistance
