# Supplementary material for: The unique C- and N-terminal sequences of Metallothionein isoform 3 mediate growth inhibition and Vectorial active transport in MCF-7 cells
Source: BMC Cancer. 2017 May 25;17:369. doi: 10.1186/s12885-017-3355-9 (PMC5445401; doi:10.1186/s12885-017-3355-9)
Supplement: Supplementary file 1 — Differential Expression Profile of MCF-7 Cells Transfected with MT1E or MT1E-CT. Table comparing gene expression profiles of MCF-7 cells transfected with the MT1E gene with MCF-7 cells transfected with MT1E-CT construct. (DOC 224 kb) [file 12885_2017_3355_MOESM4_ESM.doc]

**Differential Expression Profile of MCF-7 Cells Transfected with MT3**

**Increased Expression (pcDNA 6.2/V5Blank vs MT3)**

| **Gene ID** | **Gene Name** | **Fold Change** | **q-value (%)** | **Gene Description** | |  | |  |  |
| --- | --- | --- | --- | --- | --- | --- | --- | --- | --- |
| 1763941 | LRRC49 | 1.68154632 | 0 | leucine rich repeat containing 49 | | | |  |  |
| 1795930 | PTGER4 | 1.65058926 | 0 | prostaglandin E receptor 4 (subtype EP4) | | | | |  |
| 1793474 | INSIG1 | 1.47972609 | 0 | insulin induced gene 1 | |  | |  |  |
| 2131861 | SOCS2 | 1.39433843 | 0 | suppressor of cytokine signaling 2 | | | |  |  |
| 2117904 | ZNF22 | 1.24232022 | 0 | zinc finger protein 22 | |  | |  |  |
| 1718924 | ETFA | 1.25141209 | 0 | electron-transfer-flavoprotein, alpha polypeptide | | | | |  |
| 1685714 | INHBB | 1.28254069 | 0 | inhibin, beta B | |  | |  |  |
| 1797728 | HMGCS1 | 1.43047064 | 0 | 3-hydroxy-3-methylglutaryl-CoA synthase 1 (soluble) | | | | | |
| 2396546 | IGSF3 | 1.31676113 | 0 | immunoglobulin superfamily, member 3 | | | | |  |
| 1750324 | IGFBP5 | 1.43947267 | 0 | insulin-like growth factor binding protein 5 | | | | |  |
| 1800787 | RFTN1 | 1.52850108 | 0 | raftlin, lipid raft linker 1 | |  | |  |  |
| 1715024 | LSS | 1.31422673 | 0 | lanosterol synthase (2,3-oxidosqualene-lanosterol cyclase) | | | | | |
| 1723158 | NOP2 | 1.23491561 | 0 | NOP2 nucleolar protein | |  | |  |  |
| 1693985 | JPH1 | 1.25930190 | 0 | junctophilin 1 | |  | |  |  |
| 1755075 | IDI1 | 1.46453154 | 0.7637875 | isopentenyl-diphosphate delta isomerase 1 | | | | |  |
| 1671554 | LPIN1 | 1.43231511 | 0.7637875 | lipin 1 |  | |  |  |  |
| 1808677 | UGT2B17 | 1.33693566 | 0.7637875 | UDP glucuronosyltransferase 2 family, polypeptide B17 | | | | | |
| 1682775 | EDN1 | 1.17362193 | 0.7637875 | endothelin 1 | |  | |  |  |
| 1786065 | UHRF1 | 1.43750485 | 0.7637875 | ubiquitin-like with PHD and ring finger domains 1 | | | | |  |
| 2224143 | MCM3 | 1.17878926 | 0.7637875 | minichromosome maintenance complex component 3 | | | | | |
| 2375418 | DPH2 | 1.18459878 | 0.7637875 | DPH2 homolog | |  | |  |  |
| 1812281 | ARG1 | 1.24058157 | 0.7637875 | arginase 1 |  | |  |  |  |
| 1712888 | HSPH1 | 1.35225541 | 0.7637875 | heat shock 105kDa/110kDa protein 1 | | | |  |  |
| 1729563 | UGDH | 1.29788851 | 0.7637875 | UDP-glucose 6-dehydrogenase | | | |  |  |
| 1662932 | LCP1 | 1.18240750 | 0.7637875 | lymphocyte cytosolic protein 1 (L-plastin) | | | | |  |
| 1775192 | BCLAF1 | 1.18854234 | 1.0361676 | BCL2-associated transcription factor 1 | | | | |  |
| 2316918 | PANK1 | 1.20581089 | 1.0361676 | pantothenate kinase 1 | |  | |  |  |
| 2048793 | CIAO1 | 1.18166543 | 2.6538379 | cytosolic iron-sulfur assembly component 1 | | | | |  |
| 2336595 | ACSS2 | 1.28939926 | 2.6538379 | acyl-CoA synthetase short-chain family member 2 | | | | |  |
| 2144088 | FDFT1 | 1.25173392 | 2.6538379 | farnesyl-diphosphate farnesyltransferase 1 | | | | |  |
| 1743078 | LOC643031 | 1.43872724 | 2.6538379 | Uncharacterized |  | |  |  |  |
| 1658504 | CHKA | 1.31116962 | 2.6538379 | choline kinase alpha | |  | |  |  |
| 1662658 | PUS1 | 1.23922160 | 2.6538379 | pseudouridylate synthase 1 | | | |  |  |
| 1669113 | ATF5 | 1.35468318 | 2.6538379 | activating transcription factor 5 | | | |  |  |

**Decreased Expression (pcDNA 6.2/V5Blank vs MT3)**

|  | | |  | |  | | |  |  | | | | | |
| --- | --- | --- | --- | --- | --- | --- | --- | --- | --- | --- | --- | --- | --- | --- |
|  | **Gene ID** | **Gene Name** | | **Fold Change** | | **q-value (%)** | **Gene Description** | | | |  | |  |  |
|  | **2347798** | **IFI6** | | **0.512793** | | **0** | **interferon, alpha-inducible protein 6** | | | | | |  |  |
|  | 1725417 | NELL2 | | 0.597277 | | 0 | neural EGFL like 2 | | | |  | |  |  |
|  | 1763852 | ACACB | | 0.733247 | | 0 | acetyl-CoA carboxylase beta | | | | | |  |  |
|  | 1780058 | DEGS1 | | 0.707395 | | 0 | delta(4)-desaturase, sphingolipid 1 | | | | | |  |  |
|  | 1653292 | PFKFB4 | | 0.727643 | | 0 | 6-phosphofructo-2-kinase/fructose-2,6-biphosphatase 4 | | | | | | |  |
|  | 1770338 | TM4SF1 | | 0.572525 | | 0 | transmembrane 4 L six family member 1 | | | | | | |  |
|  | 3245682 | GAGE2B | | 0.658101 | | 0 | G antigen 2B | | | |  | |  |  |
|  | 1765796 | ENO2 | | 0.744388 | | 0 | enolase 2 (gamma, neuronal) | | | | | |  |  |
|  | 2203950 | HLA-A | | 0.578831 | | 0 | major histocompatibility complex, class I, A | | | | | | |  |
|  | 2186806 | HLA-F | | 0.720831 | | 0 | major histocompatibility complex, class I, A | | | | | | |  |
|  | 1729288 | C1QTNF6 | | 0.737452 | | 0 | C1q and tumor necrosis factor related protein 6 | | | | | | |  |
|  | 2410929 | PAPSS2 | | 0.652654 | | 0 | 3'-phosphoadenosine 5'-phosphosulfate synthase 2 | | | | | | |  |
|  | 1715638 | GAGE4 | | 0.653696 | | 0 | G antigen 4 | | |  | |  |  |  |
|  | 2138765 | PLIN2 | | 0.760120 | | 0 | perilipin 2 | | |  | |  |  |  |
|  | 2195385 | GAGE4 | | 0.629184 | | 0 | G antigen 4 | | |  | |  |  |  |
|  | 1758164 | STC1 | | 0.703155 | | 0 | stanniocalcin 1 | | | |  | |  |  |
|  | 1801377 | SLC29A4 | | 0.760713 | | 0 | solute carrier family 29 | | | |  | |  |  |
|  |  |  | |  | |  | (equilibrative nucleoside transporter), member 4 | | | | | | |  |
|  | 3243333 | GAGE12J | | 0.636249 | | 0 | G antigen 12J | | | |  | |  |  |
|  | 2294978 | RNASE4 | | 0.835982 | | 0 | angiogenin, ribonuclease, RNase A family, 5 | | | | | | |  |
|  | 2217601 | ANXA9 | | 0.737870 | | 0 | annexin A9 | | |  | |  |  |  |
|  | 1664660 | GAGE12G | | 0.644587 | | 0 | G antigen 12G | | | |  | |  |  |
|  | 3243851 | GAGE12C | | 0.637511 | | 0 | G antigen 12C | | | |  | |  |  |
|  | 1788874 | SERPINA3 | | 0.595378 | | 0 | serpin peptidase inhibitor, clade A | | | | | |  |  |
|  |  |  | |  | |  | (alpha-1 antiproteinase, antitrypsin), member 3 | | | | | | |  |
|  | 1782705 | GAGE5 | | 0.641222 | | 0 | G antigen 5 | | |  | |  |  |  |
|  | 1687384 | IFI6 | | 0.631297 | | 0 | interferon, alpha-inducible protein 6 | | | | | |  |  |
|  | 1739558 | CRELD1 | | 0.813049 | | 0 | cysteine-rich with EGF-like domains1 | | | | | |  |  |
|  | 2130441 | HLA-H | | 0.639972 | | 0 | major histocompatibility complex, class I, H (pseudogene) | | | | | | |  |
|  | 1703946 | ADORA2B | | 0.761098 | | 0 | adenosine A2b receptor | | | |  | |  |  |
|  | 1723480 | BST2 | | 0.782531 | | 0 | bone marrow stromal cell antigen 2 | | | | | |  |  |
|  | 1783832 | GAGE6 | | 0.680219 | | 0 | G antigen 6 | | |  | |  |  |  |
|  | 1776121 | MGC42367 | | 0.779823 | | 0 | KIAA1211-like | | | |  | |  |  |
|  | 2412336 | AKR1C2 | | 0.761899 | | 0 | aldo-keto reductase family 1, member C2 | | | | | | |  |
|  | 1685703 | ACOX2 | | 0.768901 | | 0 | acyl-CoA oxidase 2, branched chain | | | | | |  |  |
|  | 1742379 | IFT122 | | 0.746780 | | 0 | intraflagellar transport 122 | | | | | |  |  |
|  | 1703593 | BAIAP2L1 | | 0.825922 | | 0 | BAI1-associated protein 2-like 1 | | | | | |  |  |
|  | 2370091 | NGFRAP1 | | 0.831471 | | 0 | nerve growth factor receptor | | | | | |  |  |
|  |  |  | |  | |  | (TNFRSF16) associated protein 1 | | | | | |  |  |
|  | 1728972 | FAM64A | | 0.779616 | | 0 | family with sequence similarity 64, member A | | | | | | |  |
|  | 1721876 | TIMP2 | | 0.765500 | | 0 | TIMP metallopeptidase inhibitor 2 | | | | | |  |  |
|  | 1740466 | FAM46A | | 0.765406 | | 0 | family with sequence similarity 46, member A | | | | | | |  |
|  | 3242920 | GAGE12F | | 0.733595 | | 0 | G antigen 12F | | | |  | |  |  |
|  | 1713744 | C14orf132 | | 0.791233 | | 0 | chromosome 14 open reading frame 132 | | | | | | |  |
|  | 1769520 | UBE2L6 | | 0.671601 | | 0 | ubiquitin-conjugating enzyme E2L 6 | | | | | |  |  |
|  | 2181892 | BEX2 | | 0.827916 | | 0 | brain expressed X-linked 2 | | | | | |  |  |
|  | 1659913 | ISG20 | | 0.735738 | | 0 | interferon stimulated exonuclease gene 20kDa | | | | | | |  |
|  | 1755721 | FAM63A | | 0.770327 | | 0 | annexin A9 | | |  | |  |  |  |
|  | 2366177 | IFT122 | | 0.749090 | | 0 | intraflagellar transport 122 | | | | | |  |  |
|  | 1750974 | S100A9 | | 0.657635 | | 0 | S100 calcium binding protein A9 | | | | | |  |  |
|  | 2203729 | HCG4 | | 0.823461 | | 0 | HLA complex group 4 (non-protein coding) | | | | | | |  |
|  | 1653501 | SEMA3B | | 0.806389 | | 0 | sema domain, immunoglobulin domain (Ig), | | | | | | |  |
|  |  |  | |  | |  | short basic domain, secreted, (semaphorin) 3B | | | | | | |  |
|  | 1760727 | ANG | | 0.706914 | | 0 | angiogenin, ribonuclease, RNase A family, 5 | | | | | | |  |
|  | 1671478 | CKB | | 0.832123 | | 0 | creatine kinase, brain | | | |  | |  |  |
|  | 1742547 | NRP1 | | 0.753873 | | 0 | neuropilin 1 | | |  | |  |  |  |
|  | 1674097 | LOC645037 | | 0.668901 | | 0 | G antigen 2B | | | |  | |  |  |
|  | 1759910 | SERPINA5 | | 0.752449 | | 0 | serpin peptidase inhibitor, clade A | | | | | |  |  |
|  |  |  | |  | |  | (alpha-1 antiproteinase, antitrypsin), member 5 | | | | | | |  |
|  | 2233576 | GAGE12I | | 0.643244 | | 0 | G antigen 12I | | | |  | |  |  |
|  | 2294976 | RNASE4 | | 0.806677 | | 0 | angiogenin, ribonuclease, RNase A family, 5 | | | | | | |  |
|  | 1839750 | LOC729776 | | 0.851132 | | 0 |  | | |  | |  |  |  |
|  | 3244090 | GAGE12H | | 0.715715 | | 0 | G antigen 12H | | | |  | |  |  |
|  | 1812461 | WISP2 | | 0.587483 | | 0 | WNT1 inducible signaling pathway protein 2 | | | | | | |  |
|  | 2185984 | SASH1 | | 0.838319 | | 0 | SAM and SH3 domain containing 1 | | | | | |  |  |
|  | 1689200 | DHDH | | 0.808933 | | 0 | dihydrodiol dehydrogenase (dimeric) | | | | | |  |  |
|  | 1667748 | ANKRD33 | | 0.765466 | | 0 | ankyrin repeat domain 33 | | | | | |  |  |
|  | 1815445 | IDS | | 0.775903 | | 0 | iduronate 2-sulfatase | | | |  | |  |  |
|  | 1659766 | BAG3 | | 0.800163 | | 0 | BCL2-associated athanogene 3 | | | | | |  |  |
|  | 1758626 | IDS | | 0.775124 | | 0.958631 | iduronate 2-sulfatase | | | |  | |  |  |
|  | 1746720 | TTC39C | | 0.813604 | | 0.958631 | tetratricopeptide repeat domain 39C | | | | | |  |  |
|  | 1660691 | RAB31 | | 0.740794 | | 0.958631 | RAB31, member RAS oncogene family | | | | | | |  |
|  | 2165753 | HLA-A29.1 | | 0.610112 | | 0.958631 | major histocompatibility complex, class I, A | | | | | | |  |
|  | 1696974 | ANG | | 0.819948 | | 0.958631 | angiogenin, ribonuclease, RNase A family, 5 | | | | | | |  |
|  | 3187771 | C14orf167 | | 0.809415 | | 0.958631 | DHRS4 antisense RNA 1 | | | | | |  |  |
|  | 1745256 | CXXC5 | | 0.786875 | | 0.958631 | CXXC finger protein 5 | | | |  | |  |  |
|  | 1738450 | GAGE5 | | 0.676640 | | 0.958631 | G antigen 5 | | |  | |  |  |  |
|  | 2058782 | IFI27 | | 0.757814 | | 0.958631 | interferon, alpha-inducible protein 27 | | | | | |  |  |
|  | 1739946 | VKORC1 | | 0.833337 | | 0.958631 | vitamin K epoxide reductase complex, subunit 1 | | | | | | |  |
|  | 1808707 | FSCN1 | | 0.764591 | | 0.958631 | fascin actin-bundling protein 1 | | | | | |  |  |
|  | 1729801 | S100A8 | | 0.772787 | | 0.958631 | S100 calcium binding protein A8 | | | | | |  |  |
|  | 3283772 | LOC644237 | | 0.764125 | | 0.958631 | glyceraldehyde-3-phosphate dehydrogenase | | | | | | |  |
|  | 1693394 | BCKDK | | 0.817535 | | 0.958631 | branched chain ketoacid dehydrogenase kinase | | | | | | |  |
|  | 1745471 | IRF9 | | 0.764366 | | 0.958631 | interferon regulatory factor 9 | | | | | |  |  |
|  | 1713901 | KDELR3 | | 0.860204 | | 0.958631 | KDEL (Lys-Asp-Glu-Leu) | | | | | |  |  |
|  |  |  | |  | |  | endoplasmic reticulum protein retention receptor 3 | | | | | | |  |
|  | 2299450 | P2RX2 | | 0.792195 | | 0.958631 | purinergic receptor P2X, ligand gated ion channel, 2 | | | | | | |  |
|  | 2384056 | GPER | | 0.785665 | | 0.958631 | G protein-coupled estrogen receptor 1 | | | | | |  |  |
|  | 1698019 | LGMN | | 0.841646 | | 0.958631 | legumain | | |  | |  |  |  |
|  | 1666976 | PLD3 | | 0.718518 | | 0.958631 | phospholipase D family, member 3 | | | | | |  |  |
|  | 1744963 | ERO1L | | 0.779863 | | 0.958631 | endoplasmic reticulum oxidoreductase alpha | | | | | | |  |
|  | 2372915 | P2RY2 | | 0.834051 | | 0.958631 | purinergic receptor P2Y, G-protein coupled, 2 | | | | | | |  |
|  | 1690170 | CRABP2 | | 0.847426 | | 0.958631 | cellular retinoic acid binding protein 2 | | | | | |  |  |
|  | 1667430 | DEGS1 | | 0.664178 | | 0.958631 | delta(4)-desaturase, sphingolipid 1 | | | | | |  |  |
|  | 3248260 | LOC100132377 | | 0.838657 | | 0.958631 | Uncharacterized | | |  | |  |  |  |
|  | 1659688 | LGALS3BP | | 0.700344 | | 0.958631 | lectin, galactoside-binding, soluble, 3 binding protein | | | | | | |  |
|  | 2355831 | FHL2 | | 0.834591 | | 0.958631 | four and a half LIM domains 2 | | | | | |  |  |
|  | 3286411 | LOC644186 | | 0.791304 | | 0.958631 | synaptonemal complex central element protein 3 | | | | | | |  |
|  | 2047112 | RP11-529I10.4 | | 0.827792 | | 0.958631 | deleted in primary ciliary dyskinesia homolog (mouse) | | | | | | |  |
|  | 1794213 | ABHD14A | | 0.860892 | | 0.958631 | abhydrolase domain containing 14A | | | | | |  |  |
|  | 3289745 | LOC339352 | | 0.838645 | | 0.958631 | cytosolic thiouridylase subunit 1 | | | | | |  |  |
|  |  |  | |  | |  | homolog (S. pombe) pseudogene | | | | | |  |  |
|  | 1755643 | MGAT4A | | 0.777934 | | 0.958631 | mannosyl (alpha-1,3-)-glycoprotein | | | | | |  |  |
|  |  |  | |  | |  | beta-1,4-N-acetylglucosaminyltransferase, isozyme A | | | | | | |  |
|  | 1690653 | CDK2AP2 | | 0.807014 | | 0.958631 | cyclin-dependent kinase 2 associated protein 2 | | | | | | |  |
|  | 1768110 | ZAK | | 0.779842 | | 0.958631 | sterile alpha motif and leucine zipper containing kinase AZK | | | | | | |  |
|  | 1810836 | PDE5A | | 0.788242 | | 0.958631 | phosphodiesterase 5A, cGMP-specific | | | | | | |  |
|  | 1788421 | KCNK15 | | 0.868993 | | 0.958631 | potassium channel, two pore domain subfamily K, | | | | | | |  |
|  |  |  | |  | |  | member 15 | | |  | |  |  |  |
|  | 1755749 | PGK1 | | 0.849303 | | 0.958631 | phosphoglycerate kinase 1 | | | | | |  |  |
|  | 2359742 | CTSB | | 0.760907 | | 0.958631 | cathepsin B | | |  | |  |  |  |
|  | 2382942 | CA12 | | 0.802079 | | 0.958631 | carbonic anhydrase XII | | | |  | |  |  |
|  | 1722820 | KDELR3 | | 0.840995 | | 1.036168 | KDEL (Lys-Asp-Glu-Leu) | | | | | |  |  |
|  |  |  | |  | |  | endoplasmic reticulum protein retention receptor 3 | | | | | | |  |
|  | 1688178 | RRP7A | | 0.869726 | | 1.036168 | ribosomal RNA processing 7 homolog A | | | | | | |  |
|  | 1800739 | SPINT2 | | 0.831412 | | 1.036168 | serine peptidase inhibitor, Kunitz type, 2 | | | | | | |  |
|  | 1682326 | PCP4 | | 0.765215 | | 1.036168 | Purkinje cell protein 4 | | | |  | |  |  |
|  | 1781373 | IFIH1 | | 0.874208 | | 1.036168 | interferon induced with helicase C domain 1 | | | | | | |  |
|  | 1795298 | GPER | | 0.839880 | | 1.036168 | G protein-coupled estrogen receptor 1 | | | | | |  |  |
|  | 2390853 | CTSH | | 0.765263 | | 1.036168 | cathepsin H | | | |  | |  |  |
|  | 1715324 | HSD17B8 | | 0.772512 | | 1.036168 | hydroxysteroid (17-beta) dehydrogenase 8 | | | | | | |  |
|  | 1758066 | DSCR8 | | 0.886933 | | 1.036168 | Down syndrome critical region 8 | | | | | |  |  |
|  | 1662795 | CA2 | | 0.703978 | | 1.036168 | carbonic anhydrase II | | | |  | |  |  |
|  | 1784110 | PCTK3 | | 0.835472 | | 1.036168 | cyclin-dependent kinase 18 | | | | | |  |  |
|  | 1653200 | SLC22A17 | | 0.767355 | | 1.036168 | solute carrier family 22, member 17 | | | | | |  |  |
|  | 2077680 | CLDND2 | | 0.865464 | | 1.036168 | claudin domain containing 2 | | | | | |  |  |
|  | 1716382 | LOC387882 | | 0.818410 | | 1.036168 | chromosome 12 open reading frame 75 | | | | | | |  |
|  | 1805737 | PFKP | | 0.761561 | | 1.036168 | phosphofructokinase, platelet | | | | | |  |  |
|  | 1749109 | PSAP | | 0.830909 | | 1.036168 | prosaposin | | |  | |  |  |  |
|  | 1735979 | BCKDHA | | 0.759654 | | 1.036168 | branched chain keto acid dehydrogenase E1, | | | | | | |  |
|  |  |  | |  | |  | alpha polypeptide | | | |  | |  |  |
|  | 1804150 | HIBADH | | 0.835615 | | 1.036168 | 3-hydroxyisobutyrate dehydrogenase | | | | | |  |  |
|  | 1751956 | MGST3 | | 0.864278 | | 1.036168 | microsomal glutathione S-transferase 3 | | | | | | |  |
|  | 1745415 | BBX | | 0.785207 | | 2.653838 | bobby sox homolog (Drosophila) | | | | | |  |  |
|  | 2355559 | PSAP | | 0.841095 | | 2.653838 | prosaposin | | |  | |  |  |  |
|  | 2399300 | NAV2 | | 0.852495 | | 2.653838 | neuron navigator 2 | | | |  | |  |  |
|  | 1775016 | MPZL2 | | 0.772987 | | 2.653838 | myelin protein zero-like 2 | | | | | |  |  |

Essential genes have been bolded
