# Supplementary material for: The unique C- and N-terminal sequences of Metallothionein isoform 3 mediate growth inhibition and Vectorial active transport in MCF-7 cells
Source: BMC Cancer. 2017 May 25;17:369. doi: 10.1186/s12885-017-3355-9 (PMC5445401; doi:10.1186/s12885-017-3355-9)
Supplement: Supplementary file 2 — Differential Expression Profile of MCF-7 Cells Transfected with MT1E or MT1E-NT. Table comparing gene expression profiles of MCF-7 cells transfected with MT1E gene with MCF-7 cells transfected with MT1E-NT construct. (DOC 35 kb) [file 12885_2017_3355_MOESM1_ESM.doc]

**Differential Expression Profile of MCF-7 Cells Transfected with MT1E or MT1E-CT**

**Increased Expression (MT1E vs MT1E-CT)**

**Gene ID** **Gene Name** **Fold Change** **q-value (%)** **Gene Description**

1658885 DAGLB 1.88721412 0 Diacylglycerol lipase, beta

1681462 REG1B 1.611467878 0 Lithostatine-1-beta

**1781388 PGM5 1.303836114 0 Phosphoglucomutase-like protein 5**

**3243333 GAGE12J 1.30149099 0 G antigen 12J**

**3245682 GAGE2B 1.293480963 0 G antigen 2B**

**1783832 GAGE6 1.32444898 0 G antigen 6**

1674097 LOC645037 1.391298228 0 G antigen 1

1702806 PDCL3 1.241042479 1.16885846 Phosducin-like 3

**3244090 GAGE12H 1.276726404 1.16885846 G antigen 12H**

1741603 DBC1 1.28401913 1.16885846 Bone morphogenic protein,

**2195385 GAGE4 1.30888728 1.16885846 G antigen 4**

1747546 TSPAN1 1.209676165 1.16885846 Tetraspanin 1

**3244168 GAGE2A 1.181474622 1.16885846 G antigen 2A**

3292163 LOC391532 1.234384839 1.16885846 Uncharacterized

**1715638 GAGE4 1.328109945 1.16885846 G antigen 4**

3235517 ZNF777 1.145921609 1.16885846 Zinc finger protein 777

3176090 LOC100130919 1.246533684 1.16885846 Uncharacterized

**2132982 IGFBP5 1.310517218 1.16885846 IGF binding protein 5**

1657884 NME2 1.280876112 1.16885846 Nucleoside diphosphate kinase-2

1805519 CD24 1.178063149 1.16885846 Cell adhesion molecule 24

1683827 VPS24 1.26668107 1.16885846 Charged multivesicular body protein 3

1709590 PGM5 1.249623506 1.16885846 Phosphoglucomutase-like protein 5

1720998 CA12 1.446219062 1.16885846 Carbonic anhydrase 12

2413158 PODXL 1.189224985 1.16885846 Podocalyxin-like

2382942 CA12 1.15780102 1.16885846 Carbonic anhydrase 12

1690965 DHX9 1.224585406 1.16885846 RNA helicase A

**2233576 GAGE12I 1.355131179 1.16885846 G antigen 12I**

1751627 TRAPPC3 1.159637727 1.16885846 Trafficing protein peptide complex 3

1655849 GSC 1.138838946 1.16885846 Goosecoid homeobox

2127605 LRP3 1.240323256 1.16885846 LDL receptor-related protein 3

1707084 UBE2D4 1.136841908 1.16885846 Ubiquitin-conjugating enzyme

1678966 SNRPF 1.17612 3206 1.16885846 Small nuclear ribonuclear protein, polypeptide f

1690371 MRPL11 1.275244058 1.16885846 39s ribosomal protein L11

1726565 PIK3R2 1.324809348 1.16885846 phosphoinositol 3-kinase, beta subunit

3190570 LOC100128648 1.118440171 1.16885846 Uncharacterized

2112049 DNLZ 1.298622027 1.16885846 DNL-type zinc finger

**3243851 GAGE12C 1.311698629 1.16885846 G antigen 12C**

1803392 TAX1BP3 1.227784412 1.16885846 TAX1 binding protein 3

1750324 IGFBP5 1.280884055 3.659035178 IGF Binding Protein 5

2400874 SCYL1 1.197911225 3.659035178 Transcriptional regulator

1695962 SLC12A9 1.272856603 3.659035178 Solute transporter

1663080 LFNG 1.29801164 3.659035178 Glycosyltransferase-fucose specific

**1738450 GAGE5 1.313698379 3.659035178 G antigen 5**

**1664660 GAGE12G 1.344521324 3.659035178 G antigen 12G**

1774387 ZHX3 1.135054612 3.659035178 Zinc finger & homeobox protein 3

1741180 HEXDC 1.150614189 3.659035178 Hexosaminidase

1710752 NAPRT1 1.176110762 3.659035178 Nicotinate phosphoribosyl transferase

1813817 MRPL55 1.111802885 3.659035178 39s ribosomal protein L55

3193306 C14orf109 1.174610032 3.659035178 Transmembrane protein

1708950 ASPHD1 1.160925976 3.659035178 Aspartate β-hydroxylase domain containing 1

1698478 SNAPC2 1.191592999 3.659035178 Small nuclear RNA activating complex, polypeptide 2

**Decreased Expression (MT1E vs MT1E-CT)**

Gene ID Gene Name Fold Change q-value(%) Gene Description

1710326 CLDND1 0.836286277 0 Claudin domain-containing protein

1759097 MLLT11 0.864604067 0 Mixed lineage leukemia

1771238 CHM 0.848806201 0 Rab escort protein 1

3264543 LOC100128060 0.819320121 0 Uncharacterized

1781373 IFIH1 0.806003445 0 Interferon-induced, helicase C domain 1

2128967 C11orf1 0.83636874 0 Uncharacterized

1800626 SESN1 0.888208759 2.337716919 Sestrin 1

**1671928 PROS1 0.792101612 2.337716919 Protein S (alpha)**

1768534 BHLHB2 0.773947032 2.337716919 Basic helix-loop-helix protein 40

3278548 LOC643308 0.807076471 2.337716919 Uncharacterized

**1687384 IFI6 0.761472061 2.337716919 Interferonα-induced protein 6**

2188862 GDF15 0.802614089 2.337716919 Growth Differentiations Factor 15

2370135 HNRNPU 0.859380253 2.337716919 Heterogeneous ribonuclear protein u

2195821 C5orf41 0.824755223 2.337716919 Uncharacterized

1756311 UFSP2 0.874133049 2.337716919 UMF1-spedific peptidase 2

1766154 MRPL30 0.832182021 2.337716919 Mitochondrial ribosomal protein L30

1805800 RAB5A 0.862552573 2.337716919 Ras oncogene family

1784946 ORC3L 0.781844192 2.337716919 Origin recognition complex subunit 3

1704369 LIMA1 0.857483059 2.337716919 Lim Domain, actin binding

1695924 KLK11 0.805370572 2.337716919 Kallikrein-related peptidase 11

2329914 SPRY1 0.800737769 2.337716919 Sprouty RTK signaling antagonist 1

1788203 HEY1 0.839582339 2.337716919 Hes-related family bHLH transcription factor

1784753 PAIP2 0.841019637 2.337716919 PolyA binding protein-interacting protein 2

2415748 WSB1 0.794116619 2.337716919 SCOS box containing 1

2117904 ZNF22 0.850432634 2.337716919 Zinc finger protein 22

2047354 C1orf97 0.771854132 2.337716919 Uncharacterized

1708147 TBPL1 0.849793957 2.337716919 TBP-like 1

2095759 OGFRL1 0.805576929 2.337716919 Opioid growth factor receptor like 1

1683305 COMMD2 0.889932125 2.337716919 COOM domain containing 2

1651826 BASP1 0.770342406 2.337716919 Brain acidic soluable protein

1652989 NUP160 0.885963982 2.337716919 Nucleoprotein 160

1782609 STAG2 0.814129417 2.337716919 Stromal antigen 2

1696187 PYGL 0.830680079 2.337716919 Glycogen phosphorylase

1707506 YTHDC1 0.837629418 2.337716919 YTH domain containing protein

1843198 0.810874865 2.337716919

1752273 KIAA1143 0.861615741 2.337716919 Uncharacterized protein

1677814 ABCC3 0.840109686 2.337716919 ATP binding cassette

1748926 TMEM209 0.9004857 2.337716919 Transmembrane protein 209

1675210 ADAM22 0.802764802 2.337716919 Metalloproteinase domain 22

1790881 HNMT 0.829144649 2.337716919 Histidine methyltransferase

2071809 MGP 0.594999553 2.337716919 Matrix Gla protein

3247848 NCRNA00085 0.821942685 2.337716919 Non-protein coding RNA 85

1790650 C16orf63 0.782877581 2.337716919 Uncharacterized protein

1665510 ERRFI1 0.755942186 2.337716919 ERBB receptor feedback inhibitor

2347798 IFI6 0.696443016 2.337716919 Interferon α inducible protein 6

1755620 IER5L 0.852820086 2.337716919 Immediate response like

1767360 IL10RB 0.896205523 2.337716919 Interleukin 10 receptor β

**2269256 DNAJC12 0.830493431 2.337716919 HSP40homolog, subfam C,member12**

1707727 ANGPTL4 0.624298124 2.337716919 Angiopoietin-like 4

2261099 SORBS3 0.889676274 2.337716919 SHE containing domain 3

2168347 EPOR 0.868785011 2.337716919 Erythropoietin receptor

1732410 SLC16A9 0.883129368 3.659035178 Solute carrier, fam16, member 9

1814808 BFAR 0.842734038 3.659035178 Bifunctional apoptosis regulator

1699112 COPB1 0.792762542 3.659035178 Coalamer protein comple, β1 subunit

1708611 RDX 0.818992988 3.659035178 Radixin

2191681 CHCHD2 0.840142442 3.659035178 Coiled-coil-helix-coiled-coil-helix domain containing 2

1799082 ZNF609 0.881074154 3.659035178 Zinc finger protein 609

1681890 DYNLT3 0.76439186 3.659035178 Dynein motor family

2095840 MYST3 0.837367289 3.659035178 Histone acyltransferase family

1680453 ITM2C 0.888323213 3.659035178 Intergral membrane protein 2C

1737195 CENPK 0.742556116 3.659035178 Centromere protein K

1779258 LOC644774 0.776956048 3.659035178 Uncharacterized

1758164 STC1 0.735574085 3.659035178 Stanniocalin 1

2338038 AK3L1 0.810591067 3.659035178 Adenylate kinase 4

1803073 DNAJC12 0.823297012 3.659035178 HSP 40 homolog, subfamC, member12

2216852 PGK1 0.755829974 3.659035178 Phosphoglycerate kinase

1759387 LOC644782 0.841388857 3.659035178 Uncharacterized

1775759 NRAS 0.867402324 3.659035178 N-Ras

1759277 OIP5 0.822945136 3.659035178 Opa interacting protein 5

2125590 LOC401252 0.891297189 3.659035178 Uncharacterized

1799604 OCIAD1 0.89416323 3.659035178 OCIA domain containing 1

1695316 SLC39A8 0.821492635 3.659035178 Metal ion transporter

1755749 PGK1 0.81332589 3.659035178 Phosphoglycerate kinase

3280496 LOC100131526 0.78779189 3.659035178 Uncharacterized

1756417 ANKRD37 0.675204537 3.659035178 Ankyrin repeat domain 37

2086238 SMYD4 0.857372626 3.659035178 Set, mynd domain containing 4

2087575 ZC3H4 0.890312126 3.659035178 Zinc finger CCCH-type containing 4

1706426 DSTN 0.81835581 3.659035178 Destrin

1802355 RBM18 0.904053517 3.659035178 RNA binding motif protein 18

1675848 MYL12A 0.835962546 3.659035178 Myosin light chain 12A

1766713 HSPD1 0.802670588 3.659035178 Heat Shock Protein family D

1799688 CDC23 0.875777571 3.875688577 Cell cycle progression

1730907 ZFAND1 0.902452417 3.875688577 Zinc finger, AN-1, type 1

1688621 C9orf80 0.871677289 3.875688577 Uncharacterized

1692026 SUV420H1 0.859996222 3.875688577 histone-lysine N-methyl transferase

2058251 VIM 0.924085141 3.875688577 Vimentin

1742260 SSFA2 0.894875514 3.875688577 Sperm-specific antigen 2

2156172 HK2 0.871140537 3.875688577 Hexokinase 2

1659753 LAMP2 0.80844873 3.875688577 Lysosome associated membrane protein2

3294126 LOC100131160 0.868663888 3.875688577 Uncharacterized

Essential genes have been bolded
