# Supplementary material for: The unique C- and N-terminal sequences of Metallothionein isoform 3 mediate growth inhibition and Vectorial active transport in MCF-7 cells
Source: BMC Cancer. 2017 May 25;17:369. doi: 10.1186/s12885-017-3355-9 (PMC5445401; doi:10.1186/s12885-017-3355-9)
Supplement: Supplementary file 3 — Differential Expression Profile of MCF-7 Cells Transfected with MT3 or MT3ΔNT. Table comparing gene expression profiles of MCF-7 cells transfected with the MT3 gene with MCF-7 cells transfected with MT3ΔNT construct. (DOC 28 kb) [file 12885_2017_3355_MOESM3_ESM.doc]

**Differential Expression Profile of MCF-7 Cells Transfected with MT3 or MT3ΔNT**

**Increased Expression (MT3 vs MT3ΔNT)**

**Gene ID (ILMN)** **Gene Name** **Fold Change** **Gene Description**

2199439 CA2 1.295702 Carbonic Anhydrase II

**3243581 GAGE12C 1.405997 G Antigen 12C**

**1782705 GAGE5 1.411791 G Antigen 5**

**2233576 GAGE12I 1.421851 G Antigen 12I**

**1783832 GAGE6 1.341735 G Antigen 6**

**2195385 GAGE4 1.427266 G Antigen 4**

1720998 CA12 1.246596 Carbonic Anhydrase 12

2149226 CAV1 1.563500 Caveolin 1

**1664660 GAGE12G 1.381686 G Antigen 12G**

**3245682 GAGE2B 1.324631 G Antigen 2B**

**1738450 GAGE5 1.381862 G Antigen 5**

1793384 JAK1 1.235715 Janus Kinase 1

**3244090 GAGE12H 1.209595 G Antigen 12H**

**3243333 GAGE12J 1.329555 G Antigen 12J**

**1715638 GAGE4 1.376697 G Antigen 4**

2382942 CA12 1.187061 Carbonic Anhydrase 12

2336094 ODZ3 1.175520 Teneurin Transmembrane Protein 1

**Decreased Expression (MT3 vs MT3ΔNT)**

**Gene ID (ILMN)** **Gene Name** **Fold Change** **Gene Description**

1795930 PTGER4 0.601612 Prostaglandin E Receptor 2

Essential genes have been bolded
