# Supplementary material for: The unique C- and N-terminal sequences of Metallothionein isoform 3 mediate growth inhibition and Vectorial active transport in MCF-7 cells
Source: BMC Cancer. 2017 May 25;17:369. doi: 10.1186/s12885-017-3355-9 (PMC5445401; doi:10.1186/s12885-017-3355-9)
Supplement: Supplementary file 4 — Differential Expression Profile of MCF-7 Cells Transfected with MT3. Table comparing gene expression profiles of MCF-7 cells transfected with MT3 gene with MCF-7 cells transfected with pcDNA 6.2/V5 blank vector. (DOC 126 kb) [file 12885_2017_3355_MOESM6_ESM.doc]

**Differential Expression Profile of MCF-7 Cells Transfected with MT3ΔNT**

**Increased Expression (pcDNA 6.2/V5 BLANK vs MT3ΔNT)**

| **Gene ID** | **Gene Name** | **Fold Change** | **q-value(%)** | **Gene Description** | |  |  |  |
| --- | --- | --- | --- | --- | --- | --- | --- | --- |
| 2149226 | CAV1 | 1.73695443 | 0 | caveolin 1, caveolae protein, 22kDa | | |  |  |
| 1756572 | COQ2 | 1.253043335 | 0 | coenzyme Q2 4-hydroxybenzoate polyprenyltransferase | | | | |
| 1763941 | LRRC49 | 1.653251901 | 0 | leucine rich repeat containing 49 | | |  |  |
| 1793474 | INSIG1 | 1.345156032 | 0 | insulin induced gene 1 | |  |  |  |
| 1660372 | GRIA2 | 1.202934253 | 0 | glutamate receptor, ionotropic, AMPA 2 | | | |  |
| 1685714 | INHBB | 1.225428032 | 0 | inhibin, beta B | |  |  |  |
| 1756685 | DEPDC6 | 1.26425551 | 0 | DEP domain containing MTOR-interacting protein | | | | |
| 1735220 | CAV2 | 1.228453979 | 0 | caveolin 2 |  |  |  |  |
| 2357272 | BCLAF1 | 1.282329414 | 0 | BCL2-associated transcription factor 1 | | | |  |
| 1671554 | LPIN1 | 1.30403072 | 0 | lipin 1 |  |  |  |  |
| 1734486 | C1orf19 | 1.167399475 | 2.099868838 | TSEN15 tRNA splicing endonuclease subunit | | | |  |
| 1725981 | LOC654189 | 1.270630382 | 2.440388109 | heterogeneous nuclear ribonucleoprotein A3 | | | |  |
|  |  |  |  |  |  |  |  |  |
|  |  |  |  |  |  |  |  |  |
|  |  |  |  |  |  |  |  |  |
| **Decreased Expression (pcDNA 6.2/V5 BLANK vs MT3ΔNT)** | | | | |  |  |  |  |
|  |  |  |  |  |  |  |  |  |
| **Gene ID** | **Gene Name** | **Fold Change** | **q-value(%)** | **Gene Description** | |  |  |  |
| **2347798** | **IFI6** | **0.482271966** | **0** | **interferon, alpha-inducible protein 6** | | |  |  |
| 3242920 | GAGE12F | 0.793223457 | 0 | G antigen 12F | |  |  |  |
| 1755721 | FAM63A | 0.785508654 | 0 | annexin A9 |  |  |  |  |
| 1687384 | IFI6 | 0.535600207 | 0 | interferon, alpha-inducible protein 6 | | |  |  |
| 1730612 | DBNDD2 | 0.807843156 | 0 | dysbindin (dystrobrevin binding protein 1) | | | |  |
|  |  |  |  | domain containing 2 | |  |  |  |
| 1723480 | BST2 | 0.680346091 | 0 | bone marrow stromal cell antigen 2 | | |  |  |
| 1682567 | CCDC106 | 0.835160782 | 0 | coiled-coil domain containing 106 | | |  |  |
| 1725417 | NELL2 | 0.67093126 | 0 | neural EGFL like 2 | |  |  |  |
| 2384056 | GPER | 0.678383408 | 0 | G protein-coupled estrogen receptor 1 | | | |  |
| 1808707 | FSCN1 | 0.737097716 | 0 | fascin actin-bundling protein 1 | | |  |  |
| 2217601 | ANXA9 | 0.771579521 | 0 | annexin A9 |  |  |  |  |
| 1753342 | SAT1 | 0.786360644 | 0 | spermidine/spermine N1-acetyltransferase 1 | | | |  |
| 3286411 | LOC644186 | 0.741927814 | 0 | synaptonemal complex central element protein 3 | | | |  |
| 1795298 | GPER | 0.752145113 | 0 | G protein-coupled estrogen receptor 1 | | | |  |
| 1853876 | TMEM64 | 0.737911704 | 0 | transmembrane protein 64 | | |  |  |
| 1791147 | YPEL3 | 0.856622369 | 0 | yippee-like 3 | |  |  |  |
| 1781373 | IFIH1 | 0.853279543 | 0 | interferon induced with helicase C domain 1 | | | |  |
| 2104929 | C21orf70 | 0.870128148 | 0 | family with sequence similarity 207, member A | | | |  |
| 1780058 | DEGS1 | 0.80883057 | 2.099868838 | delta(4)-desaturase, sphingolipid 1 | | |  |  |
| 2252309 | DPP7 | 0.825683406 | 2.099868838 | dipeptidyl-peptidase 7 | |  |  |  |
| 1709043 | C9orf46 | 0.86950826 | 2.099868838 | plasminogen receptor, C-terminal lysine | | | |  |
|  |  |  |  | transmembrane protein | |  |  |  |
| 1790317 | RAB26 | 0.82606634 | 2.099868838 | RAB26, member RAS oncogene family | | | |  |
| 2150402 | TMEM64 | 0.651535799 | 2.099868838 | transmembrane protein 64 | | |  |  |
| 1811363 | NOVA1 | 0.838732472 | 2.099868838 | neuro-oncological ventral antigen 1 | | |  |  |
| 2375651 | SCNM1 | 0.86410166 | 2.099868838 | sodium channel modifier 1 | | |  |  |
| 2364674 | TRPT1 | 0.790269659 | 2.099868838 | tRNA phosphotransferase 1 | | |  |  |
| 1680110 | C10orf116 | 0.789588548 | 2.099868838 | adipogenesis regulatory factor | | |  |  |
| 1707286 | FLJ22662 | 0.80763224 | 2.099868838 | phospholipase B domain containing 1 | | | |  |
| 2058782 | IFI27 | 0.749636473 | 2.099868838 | interferon, alpha-inducible protein 27 | | | |  |
| 1721833 | IER5 | 0.835570877 | 2.099868838 | immediate early response 5 | | |  |  |
| 1774287 | CFB | 0.801952865 | 2.099868838 | complement factor B | |  |  |  |
| 2365569 | ICA1 | 0.883321835 | 2.099868838 | islet cell autoantigen 1, 69kDa | | |  |  |
| 1701918 | KLHDC9 | 0.805796354 | 2.099868838 | kelch domain containing 9 | | |  |  |
| 2116714 | SLC39A1 | 0.874942584 | 2.099868838 | solute carrier family 39 (zinc transporter), member 1 | | | | |
| 1701331 | UBE2M | 0.872498688 | 3.407334341 | ubiquitin-conjugating enzyme E2M | | |  |  |
| 1774901 | GDPD3 | 0.75706824 | 3.407334341 | glycerophosphodiester phosphodiesterase | | | |  |
|  |  |  |  | domain containing 3 | |  |  |  |
| 1800739 | SPINT2 | 0.819444566 | 3.407334341 | serine peptidase inhibitor, Kunitz type, 2 | | | |  |
| 1728972 | FAM64A | 0.823526006 | 3.407334341 | family with sequence similarity 64, member A | | | |  |
| 1749834 | LOC388588 | 0.732578579 | 3.407334341 | small integral membrane protein 1 (Vel blood group) | | | | |
| 1806030 | PPL | 0.844541562 | 3.407334341 | periplakin |  |  |  |  |
| 1681679 | TSPO | 0.77892657 | 3.407334341 | translocator protein (18kDa) | | |  |  |
| 2299450 | P2RX2 | 0.812826529 | 3.407334341 | purinergic receptor P2X, ligand gated ion channel, 2 | | | | |
| 1804955 | CTSF | 0.832740963 | 3.407334341 | cathepsin F |  |  |  |  |
| 3259146 | LOC100129681 | 0.73466894 | 3.407334341 | bone marrow stromal cell antigen 2 | | |  |  |
| 1740938 | APOE | 0.75372888 | 3.407334341 | apolipoprotein E | |  |  |  |
| 1685258 | TMEM14B | 0.868373542 | 3.407334341 | transmembrane protein 14B | | |  |  |
| 1698019 | LGMN | 0.849696627 | 3.407334341 | legumain |  |  |  |  |
| 2198185 | CXorf12 | 0.863252257 | 3.407334341 | transmembrane protein 187 | | |  |  |
| 1716382 | LOC387882 | 0.834623484 | 3.407334341 | chromosome 12 open reading frame 75 | | | |  |
| 1798952 | KDELR3 | 0.87483912 | 3.407334341 | KDEL (Lys-Asp-Glu-Leu) endoplasmic reticulum | | | | |
|  |  |  |  | protein retention receptor 3 | | |  |  |
| 1659688 | LGALS3BP | 0.70767741 | 3.407334341 | lectin, galactoside-binding, soluble, 3 binding protein | | | | |
| 1701413 | PIGQ | 0.865298739 | 3.407334341 | phosphatidylinositol glycan anchor biosynthesis, class Q | | | | |
| 1737157 | GRAMD1A | 0.853613103 | 3.407334341 | GRAM domain containing 1A | | |  |  |
| 3301740 | LOC729887 | 0.756300879 | 3.407334341 | uncharacterized LOC729887 | | |  |  |
| 2364864 | MB | 0.859044507 | 3.407334341 | myoglobin |  |  |  |  |
| 1668194 | LMTK3 | 0.755500569 | 3.407334341 | lemur tyrosine kinase 3 | |  |  |  |
| 1792508 | TMEM59 | 0.825926641 | 3.407334341 | transmembrane protein 59 | | |  |  |
| 1707127 | F2RL3 | 0.910565294 | 3.407334341 | F2R like thrombin/trypsin receptor 3 | | |  |  |
| 1719316 | TMED3 | 0.898302241 | 3.407334341 | transmembrane p24 trafficking protein 3 | | | |  |
| 2349393 | MDK | 0.73504059 | 3.407334341 | midkine (neurite growth-promoting factor 2) | | | |  |
| 3236928 | ROBLD3 | 0.850092651 | 3.407334341 | late endosomal/lysosomal adaptor, | | |  |  |
|  |  |  |  | MAPK and MTOR activator 2 | | |  |  |
| 1659766 | BAG3 | 0.861272566 | 3.407334341 | BCL2-associated athanogene 3 | | |  |  |
| 1776121 | MGC42367 | 0.826114202 | 3.407334341 | KIAA1211-like | |  |  |  |

Essential genes have been bolded
