# Supplementary material for: The unique C- and N-terminal sequences of Metallothionein isoform 3 mediate growth inhibition and Vectorial active transport in MCF-7 cells
Source: BMC Cancer. 2017 May 25;17:369. doi: 10.1186/s12885-017-3355-9 (PMC5445401; doi:10.1186/s12885-017-3355-9)
Supplement: Supplementary file 5 — Differential Expression Profile of MCF-7 Cells Transfected with MT3ΔCT. Table comparing gene expression profiles of MCF-7 cells transfected with pcDNA 6.2/V5 blank vector with MCF-7 cells transfected with MT3ΔCT construct. (DOC 698 kb) [file 12885_2017_3355_MOESM5_ESM.doc]

**Differential Expression Profile of MCF-7 Cells Transfected with MT3ΔCT**

**Increased Expression (pcDNA 6.2V5/ Blank Vector vs MT3ΔCT)**

| **Gene ID** | **Gene Name** | **Fold Change** | **q-value (%)** | **Gene Description** | |  |  |  |
| --- | --- | --- | --- | --- | --- | --- | --- | --- |
| 1715024 | LSS | 1.45099632 | 0 | lanosterol synthase | |  |  |  |
|  |  |  |  | (2,3-oxidosqualene-lanosterol cyclase) | | | |  |
| 1752923 | IFNAR1 | 1.311320837 | 0 | interferon (alpha, beta and omega) receptor 1 | | | |  |
| 1671554 | LPIN1 | 1.785373635 | 0 | lipin 1 |  |  |  |  |
| 1800787 | RFTN1 | 1.891393467 | 0 | raftlin, lipid raft linker 1 | |  |  |  |
| 1781285 | DUSP1 | 1.439491189 | 0 | dual specificity phosphatase 1 | | |  |  |
| 2371379 | ACLY | 1.402783867 | 0 | ATP citrate lyase | |  |  |  |
| 1737314 | BCL6 | 1.616860103 | 0 | B-cell CLL/lymphoma 6 | | |  |  |
| 1712537 | NODAL | 1.322688229 | 0 | nodal growth differentiation factor | | |  |  |
| 1771120 | TMEM45B | 1.317997748 | 0 | transmembrane protein 45B | | |  |  |
| 2371458 | CXCR7 | 1.649455214 | 0 | atypical chemokine receptor 3 | | |  |  |
| 1797728 | HMGCS1 | 1.5898893 | 0 | 3-hydroxy-3-methylglutaryl-CoA synthase 1 (soluble) | | | | |
| 1685714 | INHBB | 1.327832938 | 0 | inhibin, beta B | |  |  |  |
| 1788538 | NCALD | 1.370533378 | 0 | neurocalcin delta | |  |  |  |
| 3268880 | C10orf75 | 1.465903001 | 0 | oligodendrocyte maturation-associated long | | | |  |
|  |  |  |  | intergenic non-coding RNA | | |  |  |
| 3192411 | C10orf75 | 1.408964615 | 0 | oligodendrocyte maturation-associated | | | |  |
|  |  |  |  | long intergenic non-coding RNA | | |  |  |
| 2139125 | LRFN5 | 1.344450358 | 0 | leucine rich repeat and fibronectin type III | | | |  |
|  |  |  |  | domain containing 5 | |  |  |  |
| 2322498 | RORA | 1.404897527 | 0 | RAR-related orphan receptor A | | |  |  |
| 1813314 | HIST1H2BK | 1.332089058 | 0 | histone cluster 1, H2bk | |  |  |  |
| 1867517 | RORA | 1.416267029 | 0 | RAR-related orphan receptor A | | |  |  |
| 1761820 | EDARADD | 1.338521328 | 0 | EDAR-associated death domain | | |  |  |
| 2343097 | NCALD | 1.246486523 | 0 | neurocalcin delta | |  |  |  |
| 1750324 | IGFBP5 | 1.694430458 | 0 | insulin-like growth factor binding protein 5 | | | |  |
| 1793474 | INSIG1 | 1.583911001 | 0 | insulin induced gene 1 | |  |  |  |
| 1733511 | GOLGA3 | 1.33349722 | 0 | golgin A3 |  |  |  |  |
| 1797342 | FNBP1 | 1.404403022 | 0 | formin binding protein 1 | |  |  |  |
| 1793770 | DNAJB6 | 1.402459682 | 0 | DnaJ (Hsp40) homolog, subfamily B, member 6 | | | |  |
| 2311166 | ITGB5 | 1.344776795 | 0 | integrin, beta 5 | |  |  |  |
| 2404688 | NUPR1 | 1.434520326 | 0 | nuclear protein, transcriptional regulator, 1 | | | |  |
| 1674522 | HIGD1A | 1.377321436 | 0.35680157 | HIG1 hypoxia inducible domain family, member 1A | | | | |
| 2070044 | PPM1K | 1.223687557 | 0.35680157 | protein phosphatase, Mg2+/Mn2+ dependent, 1K | | | | |
| 1810431 | LOC642299 | 1.279414169 | 0.35680157 | Uncharacterized |  |  |  |  |
| 1719695 | NFKBIZ | 1.605705124 | 0.35680157 | nuclear factor of kappa light polypeptide gene | | | |  |
|  |  |  |  | enhancer in B-cells inhibitor, zeta | | |  |  |
| 2113490 | NTN4 | 1.45518082 | 0.35680157 | netrin 4 |  |  |  |  |
| 1651964 | ABCC5 | 1.290571334 | 0.62009652 | ATP-binding cassette, sub-family C (CFTR/MRP), | | | | |
|  |  |  |  | member 5 |  |  |  |  |
| 1801476 | CDS1 | 1.191140862 | 0.62009652 | CDP-diacylglycerol synthase | | |  |  |
|  |  |  |  | (phosphatidate cytidylyltransferase) 1 | | | |  |
| 2302716 | ALDH18A1 | 1.319708436 | 0.62009652 | aldehyde dehydrogenase 18 family, member A1 | | | |  |
| 1862909 | RORA | 1.577687928 | 0.62009652 | RAR-related orphan receptor A | | |  |  |
| 1730416 | CYCS | 1.267296513 | 0.62009652 | cytochrome c, somatic | |  |  |  |
| 3260286 | LOC100128062 | 1.347122449 | 0.96681715 | prenylcysteine oxidase 1 | | |  |  |
| 1769911 | SLC38A1 | 1.185053993 | 0.96681715 | solute carrier family 38, member 1 | | |  |  |
| 1654262 | ZMAT3 | 1.281923868 | 0.96681715 | zinc finger, matrin-type 3 | | |  |  |
| 1785179 | UBE2G2 | 1.289288594 | 0.96681715 | ubiquitin-conjugating enzyme E2G 2 | | |  |  |
| 2117904 | ZNF22 | 1.268176698 | 0.96681715 | zinc finger protein 22 | |  |  |  |
| 1788489 | HIST1H3F | 1.317332502 | 0.96681715 | histone cluster 1, H3f | |  |  |  |
| 1795930 | PTGER4 | 1.384463787 | 0.96681715 | prostaglandin E receptor 4 (subtype EP4) | | | |  |
| 1658144 | TRIP11 | 1.14848051 | 0.96681715 | thyroid hormone receptor interactor 11 | | | |  |
| 1775814 | GHR | 1.22880298 | 0.96681715 | growth hormone receptor | | |  |  |
| 1689156 | MMAB | 1.174833996 | 0.96681715 | methylmalonic aciduria (cobalamin deficiency) | | | |  |
|  |  |  |  | cblB type |  |  |  |  |
| 1755138 | NEK8 | 1.398920249 | 0.96681715 | NIMA-related kinase 8 | |  |  |  |
| 1668374 | ITGB5 | 1.359645394 | 0.96681715 | integrin, beta 5 | |  |  |  |
| 1792689 | HIST1H2AC | 1.433962886 | 0.96681715 | histone cluster 1, H2ac | |  |  |  |
| 2124241 | MUM1L1 | 1.225684387 | 0.96681715 | melanoma associated antigen (mutated) 1-like 1 | | | |  |
| 1713124 | AKR1C3 | 1.35231082 | 0.96681715 | aldo-keto reductase family 1, member C3 | | | |  |
| 2405305 | ARNTL | 1.356680455 | 0.96681715 | aryl hydrocarbon receptor nuclear translocator-like | | | | |
| 1730639 | SLC22A15 | 1.272607185 | 0.96681715 | solute carrier family 22, member 15 | | |  |  |
| 1840316 |  | 1.241203392 | 0.96681715 |  |  |  |  |  |
| 1810725 | FAM129A | 1.198666882 | 0.96681715 | family with sequence similarity 129, member A | | | |  |
| 1712522 | CEACAM6 | 1.348795135 | 0.96681715 | carcinoembryonic antigen-related cell adhesion molecule 6 | | | | |
|  |  |  |  | (non-specific cross reacting antigen) | | | |  |
| 2222163 | HIST1H3B | 1.193980117 | 0.96681715 | histone cluster 1, H3b | |  |  |  |
| 1819608 | ARRB1 | 1.490911593 | 0.96681715 | arrestin, beta 1 | |  |  |  |
| 1658702 | HIST1H2BJ | 1.278784403 | 0.96681715 | histone cluster 1, H2bj | |  |  |  |
| 1670752 | KIAA0907 | 1.229732804 | 0.96681715 | KIAA0907 |  |  |  |  |
| 1794392 | DDX3X | 1.165094999 | 0.96681715 | DEAD (Asp-Glu-Ala-Asp) box helicase 3, X-linked | | | | |
| 1657679 | VAV3 | 1.327050914 | 0.96681715 | vav 3 guanine nucleotide exchange factor | | | |  |
| 1810864 | PMP22 | 1.722300149 | 0.96681715 | peripheral myelin protein 22 | | |  |  |
| 1708341 | PDZK1 | 1.371165847 | 0.96681715 | PDZ domain containing 1 | | |  |  |
| 1785646 | PMP22 | 1.352588924 | 0.96681715 | peripheral myelin protein 22 | | |  |  |
| 1714445 | SLC6A9 | 1.31799855 | 0.96681715 | solute carrier family 6 | |  |  |  |
|  |  |  |  | (neurotransmitter transporter, glycine), member 9 | | | | |
| 2214117 | NDST4 | 1.319204841 | 0.96681715 | N-deacetylase/N-sulfotransferase (heparan glucosaminyl) 4 | | | | |
| 1668619 | KIAA1467 | 1.360738965 | 0.96681715 | family with sequence similarity 234, member B | | | |  |
| 1653278 | MUC20 | 1.346050801 | 1.41596843 | mucin 20, cell surface associated | | |  |  |
| 1691499 | TJP1 | 1.229748368 | 1.41596843 | tight junction protein 1 | |  |  |  |
| 1749368 | HIST1H3H | 1.387811346 | 1.41596843 | histone cluster 1, H3h | |  |  |  |
| 1743078 | LOC643031 | 1.525333296 | 1.41596843 | Uncharacterized |  |  |  |  |
| 2283325 | GPR177 | 1.414092303 | 1.41596843 | wntless Wnt ligand secretion mediator | | | |  |
| 1781691 | TRAK2 | 1.273969744 | 1.41596843 | trafficking protein, kinesin binding 2 | | |  |  |
| 1698231 | RRM2B | 1.227159256 | 1.41596843 | ribonucleotide reductase M2 B (TP53 inducible) | | | |  |
| 2336595 | ACSS2 | 1.400318866 | 1.41596843 | acyl-CoA synthetase short-chain family member 2 | | | | |
| 1661799 | HIGD1A | 1.27269701 | 1.41596843 | HIG1 hypoxia inducible domain family, member 1A | | | | |
| 1713505 | NPC1 | 1.222333851 | 1.41596843 | Niemann-Pick disease, type C1 | | |  |  |
| 1784871 | FASN | 1.433664063 | 1.41596843 | fatty acid synthase | |  |  |  |
| 2048414 | UGT2B15 | 1.418536599 | 1.41596843 | UDP glucuronosyltransferase 2 family, polypeptide B15 | | | | |
| 1677607 | SC5DL | 1.335698987 | 1.41596843 | sterol-C5-desaturase | |  |  |  |
| 1808677 | UGT2B17 | 1.562936114 | 1.41596843 | UDP glucuronosyltransferase 2 family, polypeptide B17 | | | | |
| 2276952 | TSC22D3 | 1.250521834 | 1.41596843 | TSC22 domain family, member 3 | | |  |  |
| 2383107 | VPS41 | 1.143471693 | 1.41596843 | vacuolar protein sorting 41 homolog (S. cerevisiae) | | | | |
| 3244673 | KCNU1 | 1.191824912 | 1.41596843 | potassium channel, subfamily U, member 1 | | | |  |
| 3287266 | LOC100133328 | 1.138232735 | 1.41596843 | chromosome 17 open reading frame 89 | | | |  |
| 1659990 | C7orf68 | 1.474679432 | 1.41596843 | hypoxia inducible lipid droplet-associated | | | |  |
| 2120273 | AP1S2 | 1.206016144 | 1.41596843 | adaptor-related protein complex 1, sigma 2 subunit | | | | |
| 1796755 | ITGB5 | 1.257252245 | 1.41596843 | integrin, beta 5 | |  |  |  |
| 1803036 | TARBP1 | 1.180783122 | 1.41596843 | TAR (HIV-1) RNA binding protein 1 | | | |  |
| 1793384 | JAK1 | 1.597810551 | 1.41596843 | Janus kinase 1 | |  |  |  |
| 1695422 | NCL | 1.236813087 | 1.41596843 | nucleolin |  |  |  |  |
| 1752214 | UGT2B17 | 1.277634612 | 1.41596843 | UDP glucuronosyltransferase 2 family, polypeptide B17 | | | | |
| 1756849 | HIST1H2AE | 1.303207791 | 1.41596843 | histone cluster 1, H2ae | |  |  |  |
| 1780302 | DYNC1H1 | 1.225799489 | 1.41596843 | dynein, cytoplasmic 1, heavy chain 1 | | | |  |
| 1736670 | PPP1R3C | 1.512552397 | 1.41596843 | protein phosphatase 1, regulatory subunit 3C | | | |  |
| 1670640 | ZNF664 | 1.350089503 | 1.41596843 | zinc finger protein 664 | |  |  |  |
| 1810560 | P8 | 1.291258444 | 1.41596843 | nuclear protein, transcriptional regulator, 1 | | | |  |
| 1821517 |  | 1.420941396 | 1.41596843 |  |  |  |  |  |
| 1695924 | KLK11 | 1.159538004 | 1.65196317 | kallikrein-related peptidase 11 | | |  |  |
| 1886493 | HNRNPA2B1 | 1.398243254 | 1.65196317 | heterogeneous nuclear ribonucleoprotein A2/B1 | | | |  |
| 1716816 | TMEM87A | 1.31137463 | 1.65196317 | transmembrane protein 87A | | |  |  |
| 1660436 | HSPA1B | 1.396005541 | 1.65196317 | heat shock 70kDa protein 1B | | |  |  |
| 1730773 | SNORA70 | 1.255757478 | 1.65196317 | small nucleolar RNA, H/ACA box 70 | | | |  |
| 1767351 | AR | 1.211856085 | 1.65196317 | androgen receptor | |  |  |  |
| 1720442 | NCBP2 | 1.27385368 | 1.65196317 | nuclear cap binding protein subunit 2, 20kDa | | | |  |
| 1664398 | LOC651621 | 1.212987591 | 1.65196317 | Uncharacterized |  |  |  |  |
| 1711452 | UEVLD | 1.131677917 | 1.65196317 | UEV and lactate/malate dehyrogenase domains | | | |  |
| 1714197 | ACSS2 | 1.417168172 | 1.65196317 | acyl-CoA synthetase short-chain family member 2 | | | | |
| 1686478 | HIST1H2AG | 1.302813775 | 1.65196317 | Uncharacterized |  |  |  |  |
| 1712687 | PAK2 | 1.188254857 | 1.65196317 | p21 protein (Cdc42/Rac)-activated kinase 2 | | | |  |
| 2184612 | C3orf52 | 1.215332337 | 1.65196317 | chromosome 3 open reading frame 52 | | | |  |
| 1723874 | MRPS6 | 1.228986982 | 1.65196317 | mitochondrial ribosomal protein S6 | | |  |  |
| 1664466 | KLHL9 | 1.187182537 | 1.65196317 | kelch-like family member 9 | | |  |  |
| 2075800 | PTK6 | 1.196597003 | 1.65196317 | protein tyrosine kinase 6 | | |  |  |
| 2072598 | LOC401357 | 1.15005936 | 1.65196317 | Uncharacterized |  |  |  |  |
| 1772241 | SQLE | 1.401206877 | 1.65196317 | squalene epoxidase | |  |  |  |
| 1808508 | KITLG | 1.377598472 | 1.65196317 | KIT ligand |  |  |  |  |
| 1727271 | WARS | 1.251565568 | 1.65196317 | tryptophanyl-tRNA synthetase | | |  |  |
| 1653618 | ZZZ3 | 1.184774431 | 1.65196317 | zinc finger, ZZ-type containing 3 | | |  |  |
| 1745076 | CLINT1 | 1.149765992 | 1.65196317 | clathrin interactor 1 | |  |  |  |
| 1791792 | C12orf5 | 1.194702683 | 1.65196317 | TP53 induced glycolysis regulatory phosphatase | | | |  |
| 1751120 | HIST1H4H | 1.678554936 | 1.65196317 | histone cluster 1, H4h | |  |  |  |
| 1712888 | HSPH1 | 1.56873631 | 1.65196317 | heat shock 105kDa/110kDa protein 1 | | | |  |
| 1696432 | IDH1 | 1.407486379 | 1.65196317 | isocitrate dehydrogenase 1 (NADP+), soluble | | | |  |
| 1797047 | TMEM150C | 1.297102529 | 1.65196317 | transmembrane protein 150C | | |  |  |
| 1743367 | FZD4 | 1.182297971 | 1.65196317 | frizzled class receptor 4 | |  |  |  |
| 3251501 | SERF2 | 1.167129272 | 1.65196317 | small EDRK-rich factor 2 | | |  |  |
| 1812640 | AHR | 1.210695886 | 1.65196317 | aryl hydrocarbon receptor | | |  |  |
| 1658504 | CHKA | 1.381591162 | 1.65196317 | choline kinase alpha | |  |  |  |
| 1768754 | PILRB | 1.394419707 | 1.65196317 | paired immunoglobin-like type 2 receptor beta | | | |  |
| 1801443 | TSKU | 1.220547491 | 1.65196317 | tsukushi, small leucine rich proteoglycan | | | |  |
| 1704294 | CDH3 | 1.406219468 | 1.65196317 | cadherin 3, type 1, P-cadherin (placental) | | | |  |
| 3305273 | LOC729779 | 1.309662708 | 1.65196317 | phosphoserine aminotransferase 1 | | |  |  |
| 3177285 | HNRNPR | 1.172218648 | 1.65196317 | heterogeneous nuclear ribonucleoprotein R | | | |  |
| 3278995 | LOC643167 | 1.260109438 | 1.65196317 | RNA binding motif protein 39 | | |  |  |
| 1816342 | MTRNR2L1 | 1.588145723 | 1.65196317 | MT-RNR2-like 1 | |  |  |  |
| 1797929 | MICA | 1.167607875 | 1.65196317 | MHC class I polypeptide-related sequence A | | | |  |
| 2396546 | IGSF3 | 1.176444426 | 1.65196317 | immunoglobulin superfamily, member 3 | | | |  |
| 1696485 | HNRNPAB | 1.380380506 | 1.65196317 | heterogeneous nuclear ribonucleoprotein A/B | | | |  |
| 1788416 | FAM108C1 | 1.194077839 | 1.65196317 | abhydrolase domain containing 17C | | |  |  |
| 3273854 | HNRNPA2B1 | 1.471588102 | 1.65196317 | heterogeneous nuclear ribonucleoprotein A2/B1 | | | |  |
| 1776154 | COG3 | 1.174242562 | 1.65196317 | component of oligomeric golgi complex 3 | | | |  |
| 2366703 | SGK3 | 1.21918522 | 1.65196317 | serum/glucocorticoid regulated kinase family member 3 | | | | |
| 1655694 | LOC642031 | 1.158142918 | 1.65196317 | Uncharacterized |  |  |  |  |
| 3243598 | PDZK1P1 | 1.162099824 | 1.65196317 | PDZ domain containing 1 pseudogene 1 | | | |  |
| 2224143 | MCM3 | 1.125907492 | 1.65196317 | minichromosome maintenance complex component 3 | | | | |
| 1796417 | ASNS | 1.725916607 | 2.04349989 | asparagine synthetase (glutamine-hydrolyzing) | | | |  |
| 1790354 | RABGGTB | 1.25890892 | 2.04349989 | Rab geranylgeranyltransferase, beta subunit | | | |  |
| 1712936 | C21orf66 | 1.113856502 | 2.04349989 | PAX3 and PAX7 binding protein 1 | | |  |  |
| 1679837 | SGPP1 | 1.227115092 | 2.04349989 | sphingosine-1-phosphate phosphatase 1 | | | |  |
| 1781745 | C9orf152 | 1.459214486 | 2.04349989 | chromosome 9 open reading frame 152 | | | |  |
| 1666096 | ACSL3 | 1.401145095 | 2.04349989 | acyl-CoA synthetase long-chain family member 3 | | | | |
| 1743711 | LOC650215 | 1.383474717 | 2.04349989 | exportin, tRNA | |  |  |  |
| 1677098 | YPEL2 | 1.349705628 | 2.04349989 | yippee-like 2 | |  |  |  |
| 2363621 | RBBP8 | 1.339633428 | 2.04349989 | retinoblastoma binding protein 8 | | |  |  |
| 1682139 | RAI14 | 1.329570717 | 2.04349989 | retinoic acid induced 14 | |  |  |  |
| 1849285 |  | 1.173060564 | 2.04349989 |  |  |  |  |  |
| 1676665 | CUEDC1 | 1.233685001 | 2.04349989 | CUE domain containing 1 | | |  |  |
| 1657395 | HMGCR | 1.376783973 | 2.04349989 | 3-hydroxy-3-methylglutaryl-CoA reductase | | | |  |
| 2091347 | IDH1 | 1.360357284 | 2.04349989 | isocitrate dehydrogenase 1 (NADP+), soluble | | | |  |
| 1790533 | PHACTR2 | 1.33728208 | 2.04349989 | phosphatase and actin regulator 2 | | |  |  |
| 1879480 | CCNG2 | 1.152306878 | 2.04349989 | cyclin G2 |  |  |  |  |
| 2388466 | TIA1 | 1.317551418 | 2.04349989 | TIA1 cytotoxic granule-associated RNA binding protein | | | | |
| 3283680 | LOC345041 | 1.186780449 | 2.04349989 | heat shock 60kDa protein 1 (chaperonin) | | | |  |
| 1740430 | SLC2A4RG | 1.395207519 | 2.04349989 | SLC2A4 regulator | |  |  |  |
| 1737184 | CDCA7 | 1.206015112 | 2.04349989 | cell division cycle associated 7 | | |  |  |
| 1781791 | PRRG1 | 1.136509953 | 2.04349989 | proline rich Gla (G-carboxyglutamic acid) 1 | | | |  |
| 1804629 | TPK1 | 1.372087612 | 2.04349989 | thiamin pyrophosphokinase 1 | | |  |  |
| 2157075 | LRCH4 | 1.110929972 | 2.04349989 | leucine-rich repeats and calponin homology (CH) | | | | |
|  |  |  |  | domain containing 4 | |  |  |  |
| 1763540 | PSME4 | 1.276682063 | 2.04349989 | proteasome activator subunit 4 | | |  |  |
| 1749014 | ACLY | 1.384852055 | 2.04349989 | ATP citrate lyase | |  |  |  |
| 1754969 | LMCD1 | 1.354603704 | 2.04349989 | LIM and cysteine-rich domains 1 | | |  |  |
| 1691290 | CELSR3 | 1.17297071 | 2.04349989 | cadherin, EGF LAG seven-pass G-type receptor 3 | | | | |
| 1808374 | SNTB2 | 1.212582206 | 2.04349989 | syntrophin, beta 2 (dystrophin-associated | | | |  |
|  |  |  |  | protein A1, 59kDa, basic component 2) | | | |  |
| 2211780 | SLC25A4 | 1.190493428 | 2.04349989 | solute carrier family 25 (mitochondrial carrier; | | | |  |
|  |  |  |  | adenine nucleotide translocator), member 4 | | | |  |
| 1720282 | NQO1 | 1.196429973 | 2.04349989 | NAD(P)H dehydrogenase, quinone 1 | | | |  |
| 1667030 | HSBP1 | 1.181783815 | 2.04349989 | heat shock factor binding protein 1 | | |  |  |
| 2398107 | ASNS | 1.368377869 | 2.04349989 | asparagine synthetase (glutamine-hydrolyzing) | | | |  |
| 1790689 | CRISPLD2 | 1.198899798 | 2.04349989 | cysteine-rich secretory protein LCCL | | | |  |
|  |  |  |  | domain containing 2 | |  |  |  |
| 1682783 | TUG1 | 1.173285316 | 2.04349989 | taurine up-regulated 1 (non-protein coding) | | | |  |
| 1865764 | ZMAT3 | 1.213778069 | 2.04349989 | zinc finger, matrin-type 3 | | |  |  |
| 1819515 |  | 1.086540606 | 2.04349989 |  |  |  |  |  |
| 1880280 | FOXP1-IT1 | 1.117926606 | 2.04349989 | FOXP1 intronic transcript 1 | | |  |  |
| 1720996 | SLC12A2 | 1.315327513 | 2.04349989 | solute carrier family 12 | |  |  |  |
| 1656560 | PARM1 | 1.145136955 | 2.04349989 | prostate androgen-regulated mucin-like protein 1 | | | |  |
| 1856480 |  | 1.198284718 | 2.04349989 |  |  |  |  |  |
| 1780417 | SLC25A21 | 1.107622065 | 2.04349989 | solute carrier family 25 | |  |  |  |
|  |  |  |  | (mitochondrial oxoadipate carrier), member 21 | | | |  |
| 1659544 | STX3 | 1.352355649 | 2.04349989 | syntaxin 3 |  |  |  |  |
|  |  |  |  |  |  |  |  |  |
| **Decreased Expression (pcDNA 6.2/V5 BLANK vs MT3DCT)** | | | | | |  |  |  |
|  |  |  |  |  |  |  |  |  |
| **Gene ID** | **Gene Name** | **Fold Change** | **q-value (%)** | **Gene Description** | |  |  |  |
| 3245682 | GAGE2B | 0.428312777 | 0 | G antigen 2B | |  |  |  |
| 3242920 | GAGE12F | 0.439843397 | 0 | G antigen 12F | |  |  |  |
| 1715638 | GAGE4 | 0.421677186 | 0 | G antigen 4 |  |  |  |  |
| 2195385 | GAGE4 | 0.382810566 | 0 | G antigen 4 |  |  |  |  |
| 1651958 | MGP | 0.324214944 | 0 | matrix Gla protein | |  |  |  |
| 3243333 | GAGE12J | 0.423937369 | 0 | G antigen 12J | |  |  |  |
| 1795298 | GPER | 0.469982319 | 0 | G protein-coupled estrogen receptor 1 | | | |  |
| 1725417 | NELL2 | 0.453863529 | 0 | neural EGFL like 2 | |  |  |  |
| 1664660 | GAGE12G | 0.399742315 | 0 | G antigen 12G | |  |  |  |
| 1782705 | GAGE5 | 0.409088745 | 0 | G antigen 5 |  |  |  |  |
| 1783832 | GAGE6 | 0.450212928 | 0 | G antigen 6 |  |  |  |  |
| 2071809 | MGP | 0.325037115 | 0 | matrix Gla protein | |  |  |  |
| 1738450 | GAGE5 | 0.421697334 | 0 | G antigen 5 |  |  |  |  |
| 3244090 | GAGE12H | 0.479085395 | 0 | G antigen 12H | |  |  |  |
| 1674097 | LOC645037 | 0.418563982 | 0 | G antigen 2B | |  |  |  |
| 3243851 | GAGE12C | 0.441429615 | 0 | G antigen 12C | |  |  |  |
| 2233576 | GAGE12I | 0.413867795 | 0 | G antigen 12I | |  |  |  |
| 2384056 | GPER | 0.429507773 | 0 | G protein-coupled estrogen receptor 1 | | | |  |
| **2347798** | **IFI6** | **0.573411918** | **0** | **interferon, alpha-inducible protein 6** | | |  |  |
| 2138765 | PLIN2 | 0.704539002 | 0 | perilipin 2 |  |  |  |  |
| 1674782 | LOC375295 | 0.738610818 | 0 | long intergenic non-protein coding RNA 1116 | | | |  |
| 1769520 | UBE2L6 | 0.589828702 | 0 | ubiquitin-conjugating enzyme E2L 6 | | |  |  |
| 1694502 | PRIM1 | 0.791869228 | 0 | primase, DNA, polypeptide 1 (49kDa) | | | |  |
| 2181892 | BEX2 | 0.781925888 | 0 | brain expressed X-linked 2 | | |  |  |
| 1707286 | FLJ22662 | 0.702778662 | 0 | phospholipase B domain containing 1 | | | |  |
| 3251383 | CCDC74B | 0.776131669 | 0 | coiled-coil domain containing 74B | | |  |  |
| 1768577 | PCSK6 | 0.722562524 | 0 | proprotein convertase subtilisin/kexin type 6 | | | |  |
| 1698019 | LGMN | 0.779740004 | 0 | legumain |  |  |  |  |
| 1796316 | MMP9 | 0.811147258 | 0 | matrix metallopeptidase 9 | | |  |  |
| 1713496 | ST3GAL5 | 0.704597387 | 0 | ST3 beta-galactoside alpha-2,3-sialyltransferase 5 | | | | |
| 1773337 | DKK1 | 0.646238762 | 0 | dickkopf WNT signaling pathway inhibitor 1 | | | |  |
| 1707475 | UBE2E2 | 0.750652599 | 0 | ubiquitin-conjugating enzyme E2E 2 | | |  |  |
| 1664861 | ID1 | 0.708210107 | 0 | inhibitor of DNA binding 1, dominant negative | | | |  |
|  |  |  |  | helix-loop-helix protein | |  |  |  |
| 1697499 | HLA-DRB5 | 0.819533349 | 0 | major histocompatibility complex, class II, DR beta 5 | | | | |
| 1780170 | APOD | 0.60450548 | 0 | apolipoprotein D | |  |  |  |
| 1772506 | ATP5I | 0.783417739 | 0 | ATP synthase, H+ transporting, mitochondrial Fo complex, | | | | |
|  |  |  |  | subunit E |  |  |  |  |
| 1788421 | KCNK15 | 0.820611681 | 0 | potassium channel, two pore domain subfamily K, | | | | |
|  |  |  |  | member 15 |  |  |  |  |
| 1776121 | MGC42367 | 0.752614346 | 0 | KIAA1211-like | |  |  |  |
| 3286411 | LOC644186 | 0.811395272 | 0 | synaptonemal complex central element protein 3 | | | |  |
| 1682567 | CCDC106 | 0.680346156 | 0 | coiled-coil domain containing 106 | | |  |  |
| 1678086 | CCDC74A | 0.817435328 | 0 | coiled-coil domain containing 74A | | |  |  |
| 2332964 | LGMN | 0.835021746 | 0 | legumain |  |  |  |  |
| 2058782 | IFI27 | 0.758899423 | 0 | interferon, alpha-inducible protein 27 | | | |  |
| 1709043 | C9orf46 | 0.795173439 | 0 | plasminogen receptor, C-terminal lysine | | | |  |
|  |  |  |  | transmembrane protein | |  |  |  |
| 1758066 | DSCR8 | 0.824580811 | 0 | Down syndrome critical region 8 | | |  |  |
| 1801077 | PLIN2 | 0.813489958 | 0 | perilipin 2 |  |  |  |  |
| 1747771 | LOC730744 | 0.822876992 | 0 | macrophage erythroblast attacher | | |  |  |
| 3244168 | GAGE2A | 0.67164713 | 0 | G antigen 2A | |  |  |  |
| 1666109 | MB | 0.582724229 | 0 | myoglobin |  |  |  |  |
| 1802973 | ANAPC4 | 0.851720803 | 0 | anaphase promoting complex subunit 4 | | | |  |
| 1701331 | UBE2M | 0.822444643 | 0 | ubiquitin-conjugating enzyme E2M | | |  |  |
| 2212999 | KIF5C | 0.843737743 | 0 | kinesin family member 5C | | |  |  |
| 3289745 | LOC339352 | 0.778862124 | 0 | cytosolic thiouridylase subunit 1 homolog (S. pombe) | | | | |
|  |  |  |  | pseudogene | |  |  |  |
| 1798952 | KDELR3 | 0.810026248 | 0 | KDEL (Lys-Asp-Glu-Leu) endoplasmic reticulum protein | | | | |
|  |  |  |  | retention receptor 3 | |  |  |  |
| 1728972 | FAM64A | 0.766570883 | 0 | family with sequence similarity 64, member A | | | |  |
| 1772074 | C19orf51 | 0.785919453 | 0 | dynein, axonemal, assembly factor 3 | | | |  |
| 1658040 | CRABP1 | 0.632515374 | 0 | cellular retinoic acid binding protein 1 | | | |  |
| 1682326 | PCP4 | 0.6837401 | 0 | Purkinje cell protein 4 | |  |  |  |
| 1727466 | KCNMB4 | 0.84087636 | 0 | potassium channel subfamily M regulatory beta subunit 4 | | | | |
| 1752953 | BCL2L12 | 0.766981654 | 0 | BCL2-like 12 (proline rich) | | |  |  |
| 1753143 | RHPN2 | 0.73215854 | 0 | rhophilin, Rho GTPase binding protein 2 | | | |  |
| 1723480 | BST2 | 0.72590714 | 0 | bone marrow stromal cell antigen 2 | | |  |  |
| 1780058 | DEGS1 | 0.72365205 | 0 | delta(4)-desaturase, sphingolipid 1 | | |  |  |
| 1810836 | PDE5A | 0.708972709 | 0 | phosphodiesterase 5A, cGMP-specific | | | |  |
| 1692628 | LOC652326 | 0.851059029 | 0 |  |  |  |  |  |
| 1712389 | CKLF | 0.85154374 | 0 | chemokine-like factor | |  |  |  |
| 3304898 | LOC92755 | 0.847470504 | 0 | tubulin, beta class I | |  |  |  |
| 3242105 | LOC100134073 | 0.824309368 | 0 | LY6/PLAUR domain containing 1 | | |  |  |
| 1653429 | SLC35A3 | 0.822807796 | 0 | solute carrier family 35 (UDP-N-acetylglucosamine | | | | |
|  |  |  |  | (UDP-GlcNAc) transporter), member A3 | | | |  |
| 1781742 | FAM133A | 0.870043299 | 0 | family with sequence similarity 133, member A | | | |  |
| 1803686 | ADA | 0.79201551 | 0 | adenosine deaminase | |  |  |  |
| 1713764 | LOC440928 | 0.821626624 | 0 |  |  |  |  |  |
| 1656501 | DUSP5 | 0.711869389 | 0 | dual specificity phosphatase 5 | | |  |  |
| 1796245 | DNASE2 | 0.805019354 | 0 | deoxyribonuclease II, lysosomal | | |  |  |
| 1687384 | IFI6 | 0.632029079 | 0 | interferon, alpha-inducible protein 6 | | |  |  |
| 1759910 | SERPINA5 | 0.799152527 | 0 | serpin peptidase inhibitor, clade A | | |  |  |
|  |  |  |  | (alpha-1 antiproteinase, antitrypsin), member 5 | | | |  |
| 1742379 | IFT122 | 0.757297981 | 0 | intraflagellar transport 122 | | |  |  |
| 2370091 | NGFRAP1 | 0.768784226 | 0 | nerve growth factor receptor (TNFRSF16) | | | |  |
|  |  |  |  | associated protein 1 | |  |  |  |
| 3228688 | LOC730415 | 0.799498616 | 0 |  |  |  |  |  |
| 3236963 | GAGE2E | 0.694665591 | 0 | G antigen 2E | |  |  |  |
| 1654735 | SLCO3A1 | 0.856519988 | 0 | solute carrier organic anion transporter family, | | | |  |
|  |  |  |  | member 3A1 | |  |  |  |
| 2364864 | MB | 0.659201879 | 0 | myoglobin |  |  |  |  |
| 1687821 | C16orf45 | 0.839832401 | 0 | chromosome 16 open reading frame 45 | | | |  |
| 1659688 | LGALS3BP | 0.667003795 | 0 | lectin, galactoside-binding, soluble, 3 binding protein | | | | |
| 2150402 | TMEM64 | 0.609879708 | 0.35680157 | transmembrane protein 64 | | |  |  |
| 2178226 | KRT86 | 0.686620052 | 0.35680157 | keratin 86, type II | |  |  |  |
| 1804955 | CTSF | 0.839652502 | 0.35680157 | cathepsin F |  |  |  |  |
| 1749834 | LOC388588 | 0.697441858 | 0.35680157 | small integral membrane protein 1 (Vel blood group) | | | | |
| 1673673 | PBK | 0.861727752 | 0.35680157 | PDZ binding kinase | |  |  |  |
| 1737157 | GRAMD1A | 0.800790429 | 0.35680157 | GRAM domain containing 1A | | |  |  |
| 1770338 | TM4SF1 | 0.456994425 | 0.35680157 | transmembrane 4 L six family member 1 | | | |  |
| 1660341 | LRPAP1 | 0.73632953 | 0.35680157 | low density lipoprotein receptor-related | | | |  |
|  |  |  |  | protein associated protein 1 | | |  |  |
| 1718977 | GADD45B | 0.781924763 | 0.35680157 | growth arrest and DNA-damage-inducible, beta | | | |  |
| 1663444 | LIN7B | 0.800625592 | 0.35680157 | lin-7 homolog B (C. elegans) | | |  |  |
| 3243856 | GAGE12B | 0.58517571 | 0.35680157 | G antigen 12B | |  |  |  |
| 1740466 | FAM46A | 0.677974658 | 0.35680157 | family with sequence similarity 46, member A | | | |  |
| 1810901 | RNASEH2A | 0.782754681 | 0.35680157 | ribonuclease H2, subunit A | | |  |  |
| 2305225 | NDRG4 | 0.811387701 | 0.35680157 | NDRG family member 4 | | |  |  |
| 3177532 | CECR4 | 0.865372661 | 0.35680157 | CECR5 antisense RNA 1 | | |  |  |
| 2110167 | POLR1E | 0.785085274 | 0.62009652 | polymerase (RNA) I polypeptide E, 53kDa | | | |  |
| 1708936 | EXOSC3 | 0.861811079 | 0.62009652 | exosome component 3 | |  |  |  |
| 1801442 | KRT81 | 0.541957539 | 0.62009652 | keratin 81, type II | |  |  |  |
| 1740938 | APOE | 0.728934205 | 0.62009652 | apolipoprotein E | |  |  |  |
| 2294976 | RNASE4 | 0.781724711 | 0.62009652 | angiogenin, ribonuclease, RNase A family, 5 | | | |  |
| 2294978 | RNASE4 | 0.814873833 | 0.62009652 | angiogenin, ribonuclease, RNase A family, 5 | | | |  |
| 1800739 | SPINT2 | 0.751285777 | 0.62009652 | serine peptidase inhibitor, Kunitz type, 2 | | | |  |
| 1701918 | KLHDC9 | 0.817829844 | 0.62009652 | kelch domain containing 9 | | |  |  |
| 1680110 | C10orf116 | 0.832437605 | 0.62009652 | adipogenesis regulatory factor | | |  |  |
| 3304850 | LOC730098 | 0.817052876 | 0.62009652 |  |  |  |  |  |
| 2300970 | ETFB | 0.79970457 | 0.62009652 | electron-transfer-flavoprotein, beta polypeptide | | | |  |
| 1661439 | FLOT1 | 0.82999507 | 0.62009652 | flotillin 1 |  |  |  |  |
| 2388701 | ST3GAL5 | 0.822438055 | 0.62009652 | ST3 beta-galactoside alpha-2,3-sialyltransferase 5 | | | | |
| 1677814 | ABCC3 | 0.786695745 | 0.62009652 | ATP-binding cassette, sub-family C (CFTR/MRP), | | | | |
|  |  |  |  | member 3 |  |  |  |  |
| 1853876 |  | 0.716130872 | 0.62009652 | transmembrane protein 64 | | |  |  |
| 1667114 | LOC388524 | 0.882097535 | 0.62009652 | ribosomal protein SA pseudogene 58 | | | |  |
| 3265365 | CEP78 | 0.840701974 | 0.96681715 | centrosomal protein 78kDa | | |  |  |
| 2217601 | ANXA9 | 0.764724934 | 0.96681715 | annexin A9 |  |  |  |  |
| 1774287 | CFB | 0.699492611 | 0.96681715 | complement factor B | |  |  |  |
| 1666385 | CALM3 | 0.83268225 | 0.96681715 | calmodulin 3 (phosphorylase kinase, delta) | | | |  |
| 1709590 | PGM5 | 0.850814671 | 0.96681715 | phosphoglucomutase 5 | |  |  |  |
| 1753312 | PLXDC2 | 0.852646236 | 0.96681715 | plexin domain containing 2 | | |  |  |
| 1722820 | KDELR3 | 0.798065517 | 0.96681715 | KDEL (Lys-Asp-Glu-Leu) | | |  |  |
|  |  |  |  | endoplasmic reticulum protein retention receptor 3 | | | | |
| 1755677 | FAM158A | 0.718458378 | 0.96681715 | ER membrane protein complex subunit 9 | | | |  |
| 1804384 | ASMTL | 0.804461728 | 0.96681715 | acetylserotonin O-methyltransferase-like | | | |  |
| 1775542 | FAIM3 | 0.874406741 | 0.96681715 | Fc fragment of IgM receptor | | |  |  |
| 1812461 | WISP2 | 0.397162833 | 0.96681715 | WNT1 inducible signaling pathway protein 2 | | | |  |
| 1667430 | DEGS1 | 0.657195684 | 0.96681715 | delta(4)-desaturase, sphingolipid 1 | | |  |  |
| 1762932 | CHMP2A | 0.835016006 | 0.96681715 | charged multivesicular body protein 2A | | | |  |
| 1673363 | CD97 | 0.761032398 | 0.96681715 | adhesion G protein-coupled receptor E5 | | | |  |
| 2142117 | LYPLAL1 | 0.831039493 | 0.96681715 | lysophospholipase-like 1 | | |  |  |
| 1760727 | ANG | 0.628880865 | 0.96681715 | angiogenin, ribonuclease, RNase A family, 5 | | | |  |
| 2371964 | MRPS12 | 0.828277358 | 0.96681715 | mitochondrial ribosomal protein S12 | | |  |  |
| 2203588 | MYL5 | 0.726451457 | 0.96681715 | myosin, light chain 5, regulatory | | |  |  |
| 1718046 | ARNT2 | 0.883956095 | 0.96681715 | aryl-hydrocarbon receptor nuclear translocator 2 | | | | |
| 2072178 | ECHDC3 | 0.883194322 | 0.96681715 | enoyl CoA hydratase domain containing 3 | | | |  |
| 1763638 | BCAR3 | 0.900723013 | 0.96681715 | breast cancer anti-estrogen resistance 3 | | | |  |
| 3272500 | IFI27L1 | 0.825188451 | 0.96681715 | interferon, alpha-inducible protein 27-like 1 | | | |  |
| 1735979 | BCKDHA | 0.645385615 | 0.96681715 | branched chain keto acid dehydrogenase E1, | | | |  |
|  |  |  |  | alpha polypeptide | |  |  |  |
| 1775703 | TRAPPC6A | 0.790510282 | 0.96681715 | trafficking protein particle complex 6A | | | |  |
| 2366177 | IFT122 | 0.779530934 | 0.96681715 | intraflagellar transport 122 | | |  |  |
| 1771126 | RORC | 0.892625026 | 0.96681715 | RAR-related orphan receptor C | | |  |  |
| 1713450 | MYL6B | 0.892329309 | 0.96681715 | myosin, light chain 6B, alkali, smooth muscle and non-muscle | | | | |
| 1722634 | NUCB1 | 0.734200008 | 0.96681715 | nucleobindin 1 | |  |  |  |
| 2143795 | MGC4677 | 0.795506666 | 0.96681715 | Uncharacterized |  |  |  |  |
| 1761010 | PCCB | 0.886336973 | 0.96681715 | propionyl CoA carboxylase, beta polypeptide | | | |  |
| 1674658 | COX7B2 | 0.71208269 | 0.96681715 | cytochrome c oxidase subunit VIIb2 | | | |  |
| 2412139 | CABYR | 0.813040546 | 0.96681715 | calcium binding tyrosine-(Y)-phosphorylation regulated | | | | |
| 2064725 | METTL7B | 0.805652617 | 0.96681715 | methyltransferase like 7B | | |  |  |
| 2130441 | HLA-H | 0.547621716 | 0.96681715 | major histocompatibility complex, class I, H (pseudogene) | | | | |
| 1679093 | ZNF581 | 0.664281643 | 0.96681715 | zinc finger protein 581 | |  |  |  |
| 1726603 | ATP5I | 0.798407671 | 1.41596843 | ATP synthase, H+ transporting, mitochondrial Fo complex, | | | | |
|  |  |  |  | subunit E |  |  |  |  |
| 2301083 | UBE2C | 0.747216705 | 1.41596843 | ubiquitin-conjugating enzyme E2C | | |  |  |
| 1804148 | TMED4 | 0.826686383 | 1.41596843 | transmembrane p24 trafficking protein 4 | | | |  |
| 1703946 | ADORA2B | 0.681844103 | 1.41596843 | adenosine A2b receptor | | |  |  |
| 1666976 | PLD3 | 0.709898097 | 1.41596843 | phospholipase D family, member 3 | | |  |  |
| 1669275 | ZNF16 | 0.859752872 | 1.41596843 | zinc finger protein 16 | |  |  |  |
| 2137208 | GOLT1A | 0.736819862 | 1.41596843 | golgi transport 1A | |  |  |  |
| 1666078 | HLA-H | 0.71452095 | 1.41596843 | major histocompatibility complex, class I, H (pseudogene) | | | | |
| 2198185 | CXorf12 | 0.820389618 | 1.41596843 | transmembrane protein 187 | | |  |  |
| 1719204 | PRPF31 | 0.812703049 | 1.41596843 | pre-mRNA processing factor 31 | | |  |  |
| 1678934 | POLR1E | 0.810949871 | 1.41596843 | polymerase (RNA) I polypeptide E, 53kDa | | | |  |
| 1746359 | RERG | 0.79674626 | 1.41596843 | RAS-like, estrogen-regulated, growth inhibitor | | | |  |
| 1723962 | LXN | 0.540984471 | 1.41596843 | latexin |  |  |  |  |
| 1751028 | SERPINH1 | 0.879526973 | 1.41596843 | serpin peptidase inhibitor, clade H (heat shock protein 47), | | | | |
|  |  |  |  | member 1, (collagen binding protein 1) | | | |  |
| 1781388 | PGM5 | 0.785604064 | 1.41596843 | phosphoglucomutase 5 | |  |  |  |
| 3300313 | P4HTM | 0.80501913 | 1.41596843 | prolyl 4-hydroxylase, transmembrane | | | |  |
|  |  |  |  | (endoplasmic reticulum) | | |  |  |
| 1665219 | LTBP4 | 0.863682647 | 1.41596843 | latent transforming growth factor beta binding protein 4 | | | | |
| 1730351 | FLJ35767 | 0.691889698 | 1.41596843 | testis expressed 19 | |  |  |  |
| 2117987 | TFDP1 | 0.894256958 | 1.41596843 | transcription factor Dp-1 | | |  |  |
| 1686748 | TMEM9 | 0.814147038 | 1.41596843 | transmembrane protein 9 | | |  |  |
| 1811363 | NOVA1 | 0.827533814 | 1.41596843 | neuro-oncological ventral antigen 1 | | |  |  |
| 1752967 | DHPS | 0.773940892 | 1.41596843 | deoxyhypusine synthase | | |  |  |
| 1763834 | APLP1 | 0.821211729 | 1.41596843 | amyloid beta (A4) precursor-like protein 1 | | | |  |
| 2142554 | NENF | 0.715580407 | 1.41596843 | neudesin neurotrophic factor | | |  |  |
| 1796177 | GIPC1 | 0.748332333 | 1.41596843 | GIPC PDZ domain containing family, member 1 | | | |  |
| 2130411 | KDELR1 | 0.76782265 | 1.41596843 | KDEL (Lys-Asp-Glu-Leu) | | |  |  |
|  |  |  |  | endoplasmic reticulum protein retention receptor 1 | | | | |
| 2308849 | MYADM | 0.760825827 | 1.41596843 | myeloid-associated differentiation marker | | | |  |
| 1702763 | ZMYM1 | 0.786730166 | 1.41596843 | zinc finger, MYM-type 1 | | |  |  |
| 1811470 | PLEK2 | 0.854816364 | 1.41596843 | pleckstrin 2 |  |  |  |  |
| 1777564 | MAD2L1 | 0.803521367 | 1.41596843 | MAD2 mitotic arrest deficient-like 1 (yeast) | | | |  |
| 2349393 | MDK | 0.785280927 | 1.41596843 | midkine (neurite growth-promoting factor 2) | | | |  |
| 1682930 | SIPA1 | 0.833128762 | 1.41596843 | signal-induced proliferation-associated 1 | | | |  |
| 1689059 | ZNF329 | 0.792709347 | 1.41596843 | zinc finger protein 329 | |  |  |  |
| 2413331 | TMEM107 | 0.817981807 | 1.41596843 | transmembrane protein 107 | | |  |  |
| 1703316 | LOC255783 | 0.80155482 | 1.41596843 | InaF-motif containing 1 | |  |  |  |
| 2403534 | ALOX15 | 0.843741402 | 1.41596843 | arachidonate 15-lipoxygenase | | |  |  |
| 1709486 | SRPX | 0.85744361 | 1.41596843 | sushi-repeat containing protein, X-linked | | | |  |
| 1703108 | UBE2L6 | 0.868918973 | 1.41596843 | ubiquitin-conjugating enzyme E2L 6 | | |  |  |
| 1694432 | CRIP2 | 0.772473855 | 1.41596843 | cysteine-rich protein 2 | |  |  |  |
| 1758055 | YIF1B | 0.796648027 | 1.41596843 | Yip1 interacting factor homolog B (S. cerevisiae) | | | | |
| 1755721 | FAM63A | 0.775740804 | 1.41596843 | annexin A9 |  |  |  |  |
| 2405009 | NBL1 | 0.834040202 | 1.41596843 | neuroblastoma 1, DAN family BMP antagonist | | | |  |
| 1799487 | N4BP2L1 | 0.87868596 | 1.41596843 | NEDD4 binding protein 2-like 1 | | |  |  |
| 1732612 | SHB | 0.790911999 | 1.41596843 | Src homology 2 domain containing adaptor protein B | | | | |
| 1793990 | ID2 | 0.657501195 | 1.41596843 | inhibitor of DNA binding 2, | | |  |  |
|  |  |  |  | dominant negative helix-loop-helix protein | | | |  |
| 1796423 | CLIC3 | 0.556275968 | 1.41596843 | chloride intracellular channel 3 | | |  |  |
| 1741422 | FUT8 | 0.767224846 | 1.41596843 | fucosyltransferase 8 (alpha (1,6) fucosyltransferase) | | | | |
| 1671478 | CKB | 0.755830279 | 1.41596843 | creatine kinase, brain | |  |  |  |
| 2328972 | DNMT3B | 0.871698223 | 1.41596843 | DNA (cytosine-5-)-methyltransferase 3 beta | | | |  |
| 1804798 | BEXL1 | 0.74264533 | 1.41596843 | brain expressed, X-linked 4 | | |  |  |
| 1712431 | FAM113B | 0.681759511 | 1.41596843 | PC-esterase domain containing 1B | | |  |  |
| 1671895 | ZNF613 | 0.842084418 | 1.41596843 | zinc finger protein 613 | |  |  |  |
| 1689200 | DHDH | 0.792827197 | 1.65196317 | dihydrodiol dehydrogenase (dimeric) | | | |  |
| 2165753 | HLA-A29.1 | 0.524503387 | 1.65196317 | major histocompatibility complex, class I, A | | | |  |
| 1745256 | CXXC5 | 0.774983277 | 1.65196317 | CXXC finger protein 5 | |  |  |  |
| 2252309 | DPP7 | 0.874087745 | 1.65196317 | dipeptidyl-peptidase 7 | |  |  |  |
| 2364674 | TRPT1 | 0.786774785 | 1.65196317 | tRNA phosphotransferase 1 | | |  |  |
| 1802550 | PCSK6 | 0.899996285 | 1.65196317 | proprotein convertase subtilisin/kexin type 6 | | | |  |
| 2180371 | C12orf24 | 0.721835351 | 1.65196317 | family with sequence similarity 216, member A | | | |  |
| 1657204 | SAE1 | 0.861385465 | 1.65196317 | SUMO1 activating enzyme subunit 1 | | | |  |
| 3203444 | LOC100132535 | 0.713984387 | 1.65196317 | cerebral endothelial cell adhesion molecule | | | |  |
| 1759023 | WFS1 | 0.87991775 | 1.65196317 | Wolfram syndrome 1 (wolframin) | | |  |  |
| 1805973 | GPR19 | 0.864925381 | 1.65196317 | G protein-coupled receptor 19 | | |  |  |
| 1800634 | NME4 | 0.749413782 | 1.65196317 | NME/NM23 nucleoside diphosphate kinase 4 | | | |  |
| 2234343 | ACP6 | 0.894429399 | 1.65196317 | acid phosphatase 6, lysophosphatidic | | | |  |
| 1695404 | LY6E | 0.694901272 | 1.65196317 | lymphocyte antigen 6 complex, locus E | | | |  |
| 1756326 | CKS2 | 0.699990037 | 1.65196317 | CDC28 protein kinase regulatory subunit 2 | | | |  |
| 1693270 | SUSD2 | 0.779605977 | 1.65196317 | sushi domain containing 2 | | |  |  |
| 1755643 | MGAT4A | 0.762297241 | 1.65196317 | mannosyl (alpha-1,3-)-glycoprotein beta- | | | |  |
|  |  |  |  | 1,4-N-acetylglucosaminyltransferase, isozyme A | | | |  |
| 2073184 | S1PR5 | 0.913933494 | 1.65196317 | sphingosine-1-phosphate receptor 5 | | |  |  |
| 1731699 | RAB15 | 0.81814882 | 1.65196317 | RAB15, member RAS oncogene family | | | |  |
| 1766657 | STOM | 0.839734198 | 1.65196317 | stomatin |  |  |  |  |
| 1720578 | PRAF2 | 0.908481324 | 1.65196317 | PRA1 domain family, member 2 | | |  |  |
| 2364022 | SLC16A3 | 0.780768454 | 1.65196317 | solute carrier family 16 (monocarboxylate transporter), | | | | |
|  |  |  |  | member 3 |  |  |  |  |
| 1701875 | ZYX | 0.820339009 | 1.65196317 | zyxin |  |  |  |  |
| 1655974 | PDE6B | 0.853016835 | 1.65196317 | phosphodiesterase 6B, cGMP-specific, rod, beta | | | |  |
| 2308689 | AGBL5 | 0.716980189 | 1.65196317 | ATP/GTP binding protein-like 5 | | |  |  |
| 1761560 | PHF13 | 0.894533371 | 1.65196317 | PHD finger protein 13 | |  |  |  |
| 2369826 | C16orf13 | 0.778825856 | 1.65196317 | chromosome 16 open reading frame 13 | | | |  |
| 1715169 | HLA-DRB1 | 0.83705828 | 1.65196317 | major histocompatibility complex, class II, DR beta 1 | | | | |
| 1808707 | FSCN1 | 0.681230176 | 1.65196317 | fascin actin-bundling protein 1 | | |  |  |
| 1665583 | TUBB | 0.783033829 | 1.65196317 | tubulin, beta class I | |  |  |  |
| 1789599 | NBL1 | 0.745889778 | 1.65196317 | neuroblastoma 1, DAN family BMP antagonist | | | |  |
| 1758104 | PRPS2 | 0.869913401 | 1.65196317 | phosphoribosyl pyrophosphate synthetase 2 | | | |  |
| 1691736 | ST6GALNAC6 | 0.824246915 | 1.65196317 | ST6 (alpha-N-acetyl-neuraminyl-2,3-beta-galactosyl-1,3) | | | | |
|  |  |  |  | -N-acetylgalactosaminide alpha-2,6-sialyltransferase 6 | | | | |
| 1796923 | LOC81691 | 0.769654272 | 1.65196317 | exonuclease NEF-sp | |  |  |  |
| 1813490 | FSD1 | 0.825901856 | 1.65196317 | fibronectin type III and SPRY domain containing 1 | | | | |
| 1765446 | EMP3 | 0.793252415 | 1.65196317 | epithelial membrane protein 3 | | |  |  |
| 1674874 | MFSD10 | 0.820042305 | 1.65196317 | major facilitator superfamily domain containing 10 | | | | |
| 1748427 | ZNF239 | 0.855442327 | 1.65196317 | zinc finger protein 239 | |  |  |  |
| 1688565 | ZNF580 | 0.890214099 | 1.65196317 | zinc finger protein 580 | |  |  |  |
| 2405915 | MRPS11 | 0.827915191 | 1.65196317 | mitochondrial ribosomal protein S11 | | |  |  |
| 1653200 | SLC22A17 | 0.753544565 | 1.65196317 | solute carrier family 22, member 17 | | |  |  |
| 1781281 | EPPB9 | 0.705285032 | 1.65196317 | B9 protein domain 1 | |  |  |  |
| 1657760 | SYT17 | 0.830585757 | 1.65196317 | synaptotagmin XVII | |  |  |  |
| 1688152 | IL27RA | 0.800949073 | 1.65196317 | interleukin 27 receptor, alpha | | |  |  |
| 1767481 | XRCC6BP1 | 0.890090726 | 1.65196317 | XRCC6 binding protein 1 | | |  |  |
| 1793729 | C15orf39 | 0.788964806 | 1.65196317 | chromosome 15 open reading frame 39 | | | |  |
| 1729288 | C1QTNF6 | 0.859011865 | 1.65196317 | C1q and tumor necrosis factor related protein 6 | | | |  |
| 2153916 | HSPA2 | 0.850684069 | 1.65196317 | heat shock 70kDa protein 2 | | |  |  |
| 1729650 | PEX7 | 0.818147509 | 1.65196317 | peroxisomal biogenesis factor 7 | | |  |  |
| 1696974 | ANG | 0.806748857 | 1.65196317 | angiogenin, ribonuclease, RNase A family, 5 | | | |  |
| 3242211 | TMEM187 | 0.834186218 | 1.65196317 | transmembrane protein 187 | | |  |  |
| 1671893 | CHMP2A | 0.785530636 | 1.65196317 | charged multivesicular body protein 2A | | | |  |
| 1666178 | TP53I13 | 0.86687678 | 1.65196317 | tumor protein p53 inducible protein 13 | | | |  |
| 2363668 | YIF1B | 0.723802766 | 1.65196317 | Yip1 interacting factor homolog B (S. cerevisiae) | | | | |
| 1697800 | C16orf86 | 0.890900555 | 1.65196317 | chromosome 16 open reading frame 86 | | | |  |
| 1742456 | OSTF1 | 0.875320778 | 1.65196317 | osteoclast stimulating factor 1 | | |  |  |
| 1723494 | SIRT2 | 0.835700532 | 1.65196317 | sirtuin 2 |  |  |  |  |
| 3259146 | LOC100129681 | 0.773561282 | 1.65196317 | bone marrow stromal cell antigen 2 | | |  |  |
| 1680955 | AURKA | 0.906371355 | 1.65196317 | aurora kinase A | |  |  |  |
| 1704750 | LOC647000 | 0.789204782 | 1.65196317 | tubulin, beta class I | |  |  |  |
| 1750969 | C9orf10OS | 0.846974091 | 1.65196317 | family with sequence similarity 120A opposite strand | | | | |
| 1760280 | NXT1 | 0.820147269 | 1.65196317 | nuclear transport factor 2-like export factor 1 | | | |  |
| 1719316 | TMED3 | 0.808963055 | 1.65196317 | transmembrane p24 trafficking protein 3 | | | |  |
| 1809027 | ATP5SL | 0.856151767 | 1.65196317 | ATP5S-like | |  |  |  |
| 1748206 | C20orf160 | 0.839435593 | 1.65196317 | cerebral cavernous malformation 2-like | | | |  |
| 2203950 | HLA-A | 0.487021716 | 1.65196317 | major histocompatibility complex, class I, A | | | |  |
| 2072296 | CKS2 | 0.760100347 | 1.65196317 | CDC28 protein kinase regulatory subunit 2 | | | |  |
| 1712413 | RPL39L | 0.847989844 | 1.65196317 | ribosomal protein L39-like | | |  |  |
| 1774806 | SLC9A7 | 0.891299182 | 1.65196317 | solute carrier family 9, subfamily A (NHE7, | | | |  |
|  |  |  |  | cation proton antiporter 7), member 7 | | | |  |
| 2345872 | SUMF2 | 0.816174639 | 1.65196317 | sulfatase modifying factor 2 | | |  |  |
| 1716382 | LOC387882 | 0.756853154 | 1.65196317 | chromosome 12 open reading frame 75 | | | |  |
| 1726466 | HDHD3 | 0.898483579 | 1.65196317 | haloacid dehalogenase-like hydrolase | | | |  |
|  |  |  |  | domain containing 3 | |  |  |  |
| 2382942 | CA12 | 0.831021131 | 1.65196317 | carbonic anhydrase XII | |  |  |  |
| 1732296 | ID3 | 0.523521896 | 1.65196317 | inhibitor of DNA binding 3, | | |  |  |
|  |  |  |  | dominant negative helix-loop-helix protein | | | |  |
| 3240935 | PNMA6B | 0.887245263 | 1.65196317 | paraneoplastic Ma antigen family member | | | |  |
|  |  |  |  | 6B (pseudogene) | |  |  |  |
| 2142695 | RNF4 | 0.882345697 | 1.65196317 | ring finger protein 4 | |  |  |  |
| 1699489 | TUBB6 | 0.749708311 | 1.65196317 | tubulin, beta 6 class V | |  |  |  |
| 2351638 | BEX4 | 0.797319964 | 2.04349989 | brain expressed, X-linked 4 | | |  |  |
| 1715179 | SNRPA1 | 0.845737228 | 2.04349989 | small nuclear ribonucleoprotein polypeptide A' | | | |  |
| 3238560 | IFI27L2 | 0.765422985 | 2.04349989 | interferon, alpha-inducible protein 27-like 2 | | | |  |
| 1653711 | FZD2 | 0.722822866 | 2.04349989 | frizzled class receptor 2 | |  |  |  |
| 2152402 | BAT5 | 0.87993994 | 2.04349989 | abhydrolase domain containing 16A | | |  |  |
| 1762899 | EGR1 | 0.715476654 | 2.04349989 | early growth response 1 | | |  |  |
| 3251733 | C19orf43 | 0.836673318 | 2.04349989 | chromosome 19 open reading frame 43 | | | |  |
| 1692056 | HS3ST3A1 | 0.808103605 | 2.04349989 | heparan sulfate (glucosamine) | | |  |  |
|  |  |  |  | 3-O-sulfotransferase 3A1 | | |  |  |
| 1738989 | GOLSYN | 0.802661929 | 2.04349989 | syntabulin (syntaxin-interacting) | | |  |  |
| 1669553 | UBE2E3 | 0.846750725 | 2.04349989 | ubiquitin-conjugating enzyme E2E 3 | | |  |  |
| 1702738 | KLC3 | 0.894725598 | 2.04349989 | kinesin light chain 3 | |  |  |  |
| 1768110 | ZAK | 0.765781811 | 2.04349989 | sterile alpha motif and leucine zipper containing | | | |  |
|  |  |  |  | kinase AZK | |  |  |  |
| 1681754 | GGH | 0.683944132 | 2.04349989 | gamma-glutamyl hydrolase | | |  |  |
|  |  |  |  | (conjugase, folylpolygammaglutamyl hydrolase) | | | |  |
| 1730032 | BOK | 0.784788705 | 2.04349989 | BCL2-related ovarian killer | | |  |  |
| 3284084 | LOC392522 | 0.904666372 | 2.04349989 |  |  |  |  |  |
| 1780667 | WDR51A | 0.85015801 | 2.04349989 | POC1 centriolar protein A | | |  |  |
| 1785570 | SUSD3 | 0.872750986 | 2.04349989 | sushi domain containing 3 | | |  |  |
| 1759436 | NOSIP | 0.754970877 | 2.04349989 | nitric oxide synthase interacting protein | | | |  |
| 3238148 | LOC100133435 | 0.898549594 | 2.04349989 |  |  |  |  |  |
| 2396982 | BCL2L12 | 0.787835348 | 2.04349989 | BCL2-like 12 (proline rich) | | |  |  |
| 1653599 | ATP5D | 0.752858213 | 2.04349989 | ATP synthase, H+ transporting, | | |  |  |
|  |  |  |  | mitochondrial F1 complex, delta subunit | | | |  |
| 1722533 | KATNAL1 | 0.904165823 | 2.04349989 | katanin p60 subunit A-like 1 | | |  |  |
| 1753196 | PTTG1 | 0.832259617 | 2.04349989 | pituitary tumor-transforming 1 | | |  |  |
| 1806030 | PPL | 0.839105632 | 2.04349989 | periplakin |  |  |  |  |
| 1659156 | ANKRD16 | 0.862084946 | 2.04349989 | ankyrin repeat domain 16 | | |  |  |
| 1768772 | DEGS2 | 0.748952525 | 2.04349989 | delta(4)-desaturase, sphingolipid 2 | | |  |  |
| 1688464 | MAP6D1 | 0.838218648 | 2.04349989 | MAP6 domain containing 1 | | |  |  |
| 1746948 | MYL5 | 0.72530977 | 2.04349989 | myosin, light chain 5, regulatory | | |  |  |
| 1741566 | BMP7 | 0.73533641 | 2.04349989 | bone morphogenetic protein 7 | | |  |  |
| 2355831 | FHL2 | 0.751478825 | 2.04349989 | four and a half LIM domains 2 | | |  |  |
| 2290776 | AGBL5 | 0.861323195 | 2.04349989 | ATP/GTP binding protein-like 5 | | |  |  |
| 1713285 | NAPA | 0.899500203 | 2.04349989 | N-ethylmaleimide-sensitive factor | | |  |  |
|  |  |  |  | attachment protein, alpha | | |  |  |
| 1659766 | BAG3 | 0.875944365 | 2.04349989 | BCL2-associated athanogene 3 | | |  |  |
| 1651987 | C6orf129 | 0.777790284 | 2.04349989 | coiled-coil domain containing 167 | | |  |  |
| 2415235 | CSNK1E | 0.841554924 | 2.04349989 | casein kinase 1, epsilon | |  |  |  |
| 2122420 | HMGCL | 0.824473883 | 2.04349989 | 3-hydroxymethyl-3-methylglutaryl-CoA lyase | | | |  |
| 3236928 | ROBLD3 | 0.860248269 | 2.04349989 | late endosomal/lysosomal adaptor, | | |  |  |
|  |  |  |  | MAPK and MTOR activator 2 | | |  |  |
| 1691572 | TST | 0.825827414 | 2.04349989 | thiosulfate sulfurtransferase (rhodanese) | | | |  |
| 1677080 | C11orf60 | 0.863529329 | 2.04349989 | intraflagellar transport 46 | | |  |  |
| 2175112 | KCNS3 | 0.866626563 | 2.04349989 | potassium voltage-gated channel, | | |  |  |
|  |  |  |  | modifier subfamily S, member 3 | | |  |  |
| 1657571 | ASNA1 | 0.863735922 | 2.04349989 | arsA arsenite transporter, ATP-binding, | | | |  |
|  |  |  |  | homolog 1 (bacterial) | |  |  |  |
| 1659913 | ISG20 | 0.656502235 | 2.04349989 | interferon stimulated exonuclease gene 20kDa | | | |  |
| 1681737 | TMSB15A | 0.899486373 | 2.04349989 | thymosin beta 15a | |  |  |  |
| 2238928 | RAD51C | 0.88243941 | 2.04349989 | RAD51 paralog C | |  |  |  |
| 1723678 | PRPH | 0.726810055 | 2.04349989 | peripherin |  |  |  |  |
| 2218758 | EFCBP1 | 0.848093 | 2.0435 | N-terminal EF-hand calcium binding protein 1 | | | |  |
