# Supplementary material for: The unique C- and N-terminal sequences of Metallothionein isoform 3 mediate growth inhibition and Vectorial active transport in MCF-7 cells
Source: BMC Cancer. 2017 May 25;17:369. doi: 10.1186/s12885-017-3355-9 (PMC5445401; doi:10.1186/s12885-017-3355-9)
Supplement: Supplementary file 6 — Differential Expression Profile of MCF-7 Cells Transfected with MT3ΔNT. Table comparing gene expression profiles of MCF-7 cells transfected with pcDNA 6.2/V5 blank vector with MCF-7 cells transfected with MT3ΔNT construct. (DOC 28 kb) [file 12885_2017_3355_MOESM2_ESM.doc]

**Differential Expression Profile of MCF-7 Cells Transfected with MT1E or MT1ENT**

**Increased Expression (MT1E vs MT1E-NT)**

**Gene ID** **Gene Name** **Fold Change** **q-value(%)** **Gene Description**

1763941 LRRC49 2.228437 0 Leucine rich repeat 49

**2132982 IGFBP5 1.509016 0 IGF binding protein 5**

1732296 ID3 1.516312 0 Inhibitor of DNA binding 3

1796423 CLIC3 1.241121 0 Chloride intracellular channel 3

**1750324 IGFBP5 1.519913 0 IGF binding protein 5**

2150258 ZFP36L2 1.378217 0 Ring finger binding protein-like 2

1664861 ID1 1.330076 0 Inhibitor of DNA binding 1

1811624 THADA 1.181123 0 Thyroid adenoma associated

1779416 SCUBE2 1.405292 0 Signal peptide, EGF-like 2, CUB domain

1741566 BMP7 1.740043 0 Bone morphogenic protein 7

1757440 FAM69B 1.282523 0 Sequence similarity 69, member B

1793990 ID2 1.411532 0 Inhibitor of DNA binding 2

3180855 LOC100128077 1.217154 0 Uncharacterized

1707339 BTG3 1.271313 1.524301 BTG family member 3

1760412 SHISA2 1.365835 1.524301 Shisa family member 2

**1781388 PGM5 1.287049 1.524301 Phosphoglucomutase 5**

1676663 TNFRSF11B 1.348879 2.104987 Tumor necrosis factor receptor superfamily, 11b

2131861 SOCS2 1.362049 2.104987 Suppressor of cytokine signaling 2

2149226 CAV1 1.281969 8.595362 Caveolin 1

2136147 BCAS1 1.237293 8.595362 Breast carcinoma amplified sequence 1

1713995 SCNN1A 1.297634 8.595362 sodium channel, non voltage gated 1 alpha subunit

**Decrease Expression (MT1E vs MT1E-NT)**

Gene ID Gene Name Fold Change q-value(%) Gene Description

**3245682 GAGE2B 0.466329604 0 G antigen 2B**

**3243333 GAGE12J 0.459488705 0 G antigen 12J**

1674097 LOC645037 0.467454787 0 Uncharacterized

2071809 MGP 0.336357278 0 Matrix Gla protein

**3244168 GAGE2A 0.66439384 0 G antigen 2A**

**3244090 GAGE12H 0.498403963 0 G antigen 12H**

1651958 MGP 0.379721264 0 Matrix Gla protein

**2233576 GAGE12I 0.468604803 0 G antigen 12I**

**1783832 GAGE6 0.483057839 0 G antigen 6**

**3243851 GAGE12C 0.485792506 0 G antigen 12C**

**1715638 GAGE4 0.474997117 0 G antigen 4**

**3242920 GAGE12F 0.496778402 0 G antigen 12F**

**2195385 GAGE4 0.457295766 0 G antigen 4**

**1664660 GAGE12G 0.453022047 0 G antigen 12G**

3307002 TMEM83 0.516792024 0 non-protein coding RNA 52

**1782705 GAGE5 0.473479596 0 G antigen 5**

**1738450 GAGE5 0.478769694 0 G antigen 5**

**3236963 GAGE2E 0.65044128 0 G antigen 2E**

**3243856 GAGE12B 0.598389289 0 G antigen 12B**

1682326 PCP4 0.758998507 0 Purkinje cell protein 4

1770978 TMEM83 0.500088657 0 non-protein coding RNA 52

**1803073 DNAJC12 0.845289163 0 Hsp 40 homolog subfamily C, member12**

**3237846 GAGE12E 0.786027973 0 G antigen 12E**

2395451 ASS1 0.706032222 0 Argininosuccinate synthase 1

1853876 TMEM64 0.808576845 0 Transmembrane protein 64

**2347798 IFI6 0.669914591 0 interferon, alpha-inducible protein 6**

**1687384 IFI6 0.717891454 0 interferon, alpha-inducible protein 6**

1703110 KCNE4 0.85134947 0 Potassium channel, beta subunit

1781745 C9orf152 0.646063275 0 Ch 9 Open reading frame 152

1764754 RAMP1 0.794885458 0 receptor activity modifying protein (G protein coupled)

1729801 S100A8 0.720883843 0 S100 calcium binding protein A8

**2269256 DNAJC12 0.821496534 0 Hsp 40 homolog, subfamily C, member12**

1716843 ELOVL2 0.779843799 0 ELOVL fatty acid elongase 2

1675210 ADAM22 0.792171298 0 ADAM metallopeptidase domain 22

1764321 ACOT4 0.846216662 0 acyl-CoA thioesterase 4

2193980 ABCB6 0.862551353 0 ATP Binding Cassette

1741159 MAP3K8 0.813464281 0 Mitogen activated protein kinase kinase kinase 8

1763852 ACACB 0.844525171 0 Acetyl-CoA carboxylase beta

1708778 ASS1 0.725171305 0 argininosuccinate synthase 1

2246956 BCL2 0.771915498 1.524301 B-cell CLL/lymphoma2

1690217 BFSP2 0.720380463 1.524301 phakinin

1668619 KIAA1467 0.829634324 1.524301 sequence similarity 234, member 8

1732071 HIST2H2BE 0.81174528 2.104987 histone cluster 2, H2be

1762606 AQP11 0.899181144 2.104987 aquaporin 11

1701077 LOC642897 0.848946745 2.104987 N-6 adenine-specific DNA methyltransferase 1

2349393 MDK 0.868326639 2.104987 Midkine (neurite growth promoting factor 2

**1671928 PROS1 0.8645277 8.595362 Protein S (alpha)**

2160210 TACSTD1 0.877911843 8.595362 Epitheilial cell adhersion molecule

1750974 S100A9 0.745653456 8.595362 S100 calcium binding protein

2230862 GYG1 0.874277007 8.595362 Glycogenin 1

Essential genes have been bolded
